# Self-Diffusive Properties of the Intrinsically Disordered Protein Histatin 5 and the Impact of Crowding Thereon: A Combined Neutron Spectroscopy and Molecular Dynamics Simulation Study

**DOI:** 10.1021/acs.jpcb.1c08976

**Published:** 2022-01-19

**Authors:** Eric Fagerberg, Samuel Lenton, Tommy Nylander, Tilo Seydel, Marie Skepö

**Affiliations:** † Theoretical Chemistry, Lund University, POB 124, SE-221 00 Lund, Sweden; ‡ Physical Chemistry, Lund University, POB 124, SE-221 00 Lund, Sweden; ¶ Institut Max von Laue - Paul Langevin, 71 avenue des Martyrs, CS 20156, F-38042 Grenoble, France; § LINXS - Lund Institute of Advanced Neutron and X-ray Science, Scheelevägen 19, SE-223 70 Lund, Sweden

## Abstract

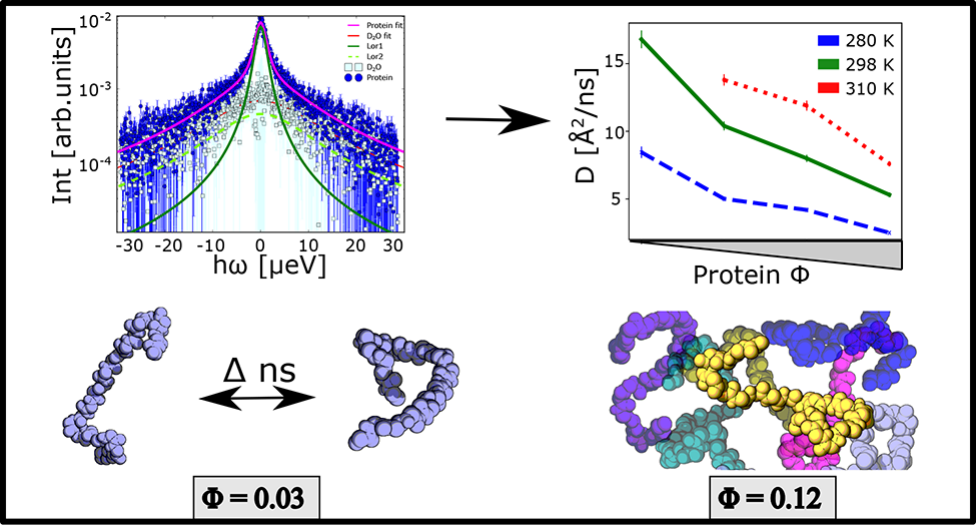

Intrinsically disordered proteins (IDPs) are proteins that, in comparison with globular/structured proteins, lack a distinct tertiary structure. Here, we use the model IDP, Histatin 5, for studying its dynamical properties under self-crowding conditions with quasi-elastic neutron scattering in combination with full atomistic molecular dynamics (MD) simulations. The aim is to determine the effects of crowding on the center-of-mass diffusion as well as the internal diffusive behavior. The diffusion was found to decrease significantly, which we hypothesize can be attributed to some degree of aggregation at higher protein concentrations, (≥100 mg/mL), as indicated by recent small-angle X-ray scattering studies. Temperature effects are also considered and found to, largely, follow Stokes–Einstein behavior. Simple geometric considerations fail to accurately predict the rates of diffusion, while simulations show semiquantitative agreement with experiments, dependent on assumptions of the ratio between translational and rotational diffusion. A scaling law that previously was found to successfully describe the behavior of globular proteins was found to be inadequate for the IDP, Histatin 5. Analysis of the MD simulations show that the width of the distribution with respect to diffusion is not a simplistic mirroring of the distribution of radius of gyration, hence, displaying the particular features of IDPs that need to be accounted for.

## 1 Introduction

In contrast to globular proteins, intrinsically disordered proteins (IDPs) lack a well-defined three-dimensional structure, instead they adopt an ensemble of conformers in solution.^1^ Consequently, IDPs can rapidly sample a large volume of conformational space.^2^ This innate flexibility, combined with the ability of IDPs to bind with high specificity, allows a single IDP to regulate a range of biological functions.^3^

The precise nature of the conformational ensembles adopted by IDPs depend on a variety of conditions including, for example, temperature, ionic strength, and presence of binding partners.^4,5^ One condition, often neglected by experimental studies, is the effect of crowding on the dynamical properties of IDPs, and how these effects relate to the protein function. Determining the dynamical properties under crowded conditions is pertinent due to the high intracellular concentration of macromolecules, which can reach up to 400 mg/mL.^6,7^ At these concentrations, it is expected that protein–protein interactions, as excluded volume effects and electrostatic interactions, impact not only the conformational ensemble,^8−10^ but also restrict the ability of IDPs to diffuse throughout the crowded intracellular milieu.^11^

Both these factors have important implications for how the functions of IDPs are regulated intracellularly. For example, a reduction in diffusion caused by macromolecular crowding could provide spatial means of controlling IDP interactions, while reducing the flexibility of the conformational ensemble may restrict the rate at which IDPs can interact with other macromolecules.^12^ In order to study the diffusive properties of IDPs under crowded conditions, appropriate time and length scales must be considered. The diffusive dynamics of IDPs take place on hierarchical time and length-scales covering a range from picoseconds to hours and from ångströms to micrometers.^13^

Due to both the high concentrations of proteins required to accurately represent intracellular conditions, and the relatively small spatial and temporal scales on which the diffusive motions of IDPs take place, studying these properties experimentally is not straightforward. Measurements of the translational diffusion of an IDP and a globular protein in crowded environments have been completed by NMR on relatively long time-scales. Wang et al. determined that under crowded conditions, the IDP had faster translational diffusion compared to the globular protein, while under dilute conditions the opposite was observed.^14^

Similarly a single-molecule experiment, completed in cells, has shown that the diffusion of an IDP is faster than a globular protein (while both have significantly reduced diffusion) upon crowding and that this effect is largely based on the size of the crowding molecule.^15^ Despite the insights provided by such experimental studies, the nanosecond time scale of translational diffusion is difficult to access, leaving this time window of IDP diffusion under crowded conditions largely unexplored.

In recent years, significant advancements have been made in the instrumentation of high-resolution neutron spectroscopy, increasing the possibilities for studying biological samples using this technique. Parallel developments in the analysis methods used to deconvolute the resulting experimental data means that it is now possible to simultaneously measure the center-of-mass (COM) and their superimposed internal diffusive motions of proteins in solution, on the nanosecond time scale, and the ångström length scale. In contrast to, for example, fluorescence spectroscopy, these methods have the advantages of being label-free, having access to shorter time scales and allowing for the gathering of simultaneous spatial and dynamical information.^16^ Neutron spectroscopy also complements NMR that accesses angular correlations and generally longer times scales.^17^ Moreover, neutron techniques provide access to opaque samples such as highly turbid protein solutions that are difficult to measure by, e.g., light scattering. Numerous studies investigating the diffusion of globular proteins in solution using these novel neutron methods have been published during the last few years.^18−22^ Similar studies of IDPs under crowded conditions are, however, comparatively rare.^23^

On the nanosecond observation time scale, and nanometer observation length scale of high-resolution quasi-elastic neutron spectroscopy (QENS), the observable COM diffusion coefficient *D* = *D*(*D*_*r*_, *D*_*t*_) consists of contributions from both rotational *D*_*r*_ and translational *D*_*t*_ diffusion, and corresponds to the so-called short-time diffusion in terms of the physics of colloidal hard sphere suspensions.^19,24^ When native proteins rich in hydrogen atoms are suspended in a deuterated solvent, the measured spectra from the proteins possess the information on the self-, or, synonymously, tracer diffusion of these proteins due to the prevailing incoherent scattering from the proteins. On the diffusive short-time scale, the proteins diffuse on average by only a small fraction of their hydrodynamic radius. Therefore, their diffusion is governed by hydrodynamic interactions, while direct interactions, i.e., collisions, can be neglected. An advantage of probing self-diffusion consists in an unambiguous access to the hydrodynamic size of the diffusing assembly, since no effect from the static structure needs to be taken into account.^22,25^ Moreover, the prevailing incoherent scattering allows in principle to determine the elastic incoherent structure factors (EISF) of the proteins in solution, which provides information on the geometry of the confinement of the internal diffusive motions.^25,26^ For dilute protein solutions, the EISF can be measured only since recently, employing spectrometers with the highest neutron beam brightness and signal-to-noise ratio, while previous neutron spectroscopy studies have already explored IDPs and globular proteins in hydrated powder states.^27−29^ A challenge for IDPs, as opposed to, e.g., globular proteins with a stable structure, is due to their fluctuating shape which may result in a fluctuating hydrodynamic radius as well as possibly a fluctuating EISF. Moreover, the possibly more open average shape of IDPs may render any picture of compact colloidal objects insufficient to describe IDPs.

Here, we employ high-resolution neutron backscattering spectroscopy accessing high momentum transfers typically within 0.2 Å^–1^ < *q* < 2.0 Å^–1^ to probe the self-diffusion of the extensively studied, relatively short, IDP, Histatin 5 (Hst5).^30−34^ We first discuss the experimental results from high-resolution QENS on aqueous solutions of Hst5 in terms of the established models for well-folded proteins with a compact shape.^16,35^ Hst5 has been well-investigated in terms of structure with SAXS,^36,37^ NMR,^38,39^ and circular dichroism,^40,41^ including investigations on the effect of temperature, crowding, and to limited extent salt, often combined with simulation to further interpret results or benchmark simulation models.^42−46^

Thereafter we compare the experimental results with full atomistic molecular dynamics simulations from which both the COM diffusive dynamics and the EISF are obtained. Based on this comparison, we discuss the significant impact of the fluctuating shape of the IDPs on the nanosecond observation time scale of our experiment, and comparisons are made with globular proteins.

## 2 Methods

### 2.1 Sample Preparation

Hst5 was purchased from Genemed Synthesis, Inc. (San Antonio, USA) and TAG Copenhagen A/S (Copenhagen, Denmark). In case of nondialyzed samples, the protein was used directly as obtained. The protein concentration was determined with a Thermo Scientific Nanodrop OneC UV–vis spectrophotometer using an extinction coefficient of ϵ = 2560 M^–1^ cm^–1^, and molecular weight of 3.036 kDa. The buffer used contained D_2_O with 20 mM Tris, with either 150 mM NaCl or nominally 10 mM NaCl (no salt was explicitly added; only a small amount of sodium hydroxide required to set the pH to 7 contributes to the salt content in this case). In case of the dialyzed samples, 6–8 cm long pieces of 16 mm flat width, 500–1000 MWCO membranes (SpectrumLabs, Piraeus, Greece), were used to dialyze the protein. The protein powder was taken from the can and dissolved in Milli-Q water, and exhaustively dialyzed against Milli-Q water, at least 200 times its volume with four water changes. Between the water changes, the dialysis was left to proceed for 4–12 h at room temperature. After dialysis, the protein was freeze-dried and stored at −20 °C. Thereafter, the sample preparation procedure was the same as for nondialyzed samples. All samples measured are found in Table S1.

### 2.2 QENS

The QENS experiments^47−49^ were carried out on the backscattering spectrometer IN16B at the Institut Laue-Langevin, Grenoble, France.^50,51^ A scattering vector range of 0.2 < *q* < 1.8 Å^–1^ was covered, using Si111 monochromator and analyzers, corresponding to an elastic neutron wavelength of 6.271 Å. QENS spectra were recorded by mechanically Doppler-shifting the incident energy through a movement of the monochromator. Examples of these spectra are found in Figures S1 and S2 in the Supporting Information.

### 2.3 Analysis

The QENS data were reduced using *Mantid*(52) and subsequently fitted using python scripts derived from https://github.com/seydel/QENS_utilities.

The scattering function *S*(*q*, ω) recorded on IN16B depends on the momentum transfer *ℏq* and energy transfer *ℏω*, and was modeled by eq 1,^16^
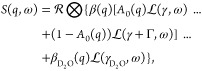
1where  denotes the spectrometer resolution function, *A*_0_(*q*) the EISF, and  a Lorentzian function. β(*q*) and  represent scalars. The Lorentzian widths γ, Γ, and  account for the contributions from the protein COM diffusion, superimposed internal protein diffusion, and solvent water diffusion, respectively.

Importantly, the fits can be performed with and without imposing a dependence of the model (eq 1) on the momentum transfer *q*, respectively. For the COM diffusion, Fickian-type diffusion has been confirmed for numerous well-folded proteins,^16^
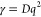
2with the observable apparent COM diffusion coefficient *D*. The internal diffusive motions of the proteins have previously been described by the simplistic jump-diffusion model,^16,35^ which has proven sufficient on the rather narrow energy range of −30 μeV ≤ *ℏω* ≤ + 30 μeV covered by our present experiment,
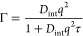
3with the internal diffusion coefficient *D*_int_ and the residence time between diffusive jumps τ. On the observation time scale of our experiments, it is assumed that eq 3 approximately accounts for protein backbone fluctuations, while side-chain motions are too fast to be captured.^16^ In this work, two fitting approaches are compared, both based on eq 1: The first approach is to fit the spectra obtained for each value of *q* separately (“per-*q*”) and to subsequently fit eq 2 to the obtained γ(*q*). The second approach consists of imposing both eq 2 for COM and eq 3 for internal diffusivity in a fit of the spectra for all *q* simultaneously, denoted “jump-diffusion” fit.

According to Cragnell et al.,^36^ the specific volume, ν_*p*_, of Hst5 is 0.702 3 mL/g, calculated with Sednterp, which uses a method by Cohn and Edsall.^53^ The volume fractions can then be determined,
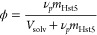
4with *V*_*solv*_ being the volume of the solvent. The equation can be rearranged to be expressed with concentrations.
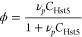
5

The fit was evaluated with the goodness-of-fit, L1 loss function, and L2 loss function. For goodness-of-fit,
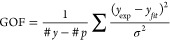
6where #*y* is the number of experimental data points, #*p* is the number of parameters, *y*_exp_ is the experimental data, y_fit_ is the fitted data and σ is the experimental error. For the L1 loss function,
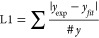
7and the L2 loss function.
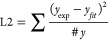
8

If singular observations are poorly fitted altough the overall fits are reasonable, this would be captured by comparing the L1 and L2 loss functions. Viscosities of pure water and of deuterium oxide were calculated according to the relation by Cho et al.^54^ To account for how changes in solvent properties affect protein behavior, the Stokes–Einstein equation is used to normalize the data for temperature and viscosity, yielding an effective hydrodynamic radius (*R*_*eff*_),
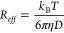
9as the diffusion achieved from QENS is an apparent diffusion, rather than a translational diffusion *D*_*t*_.

#### Paalman–Pings Correction, Jump-Diffusion vs per-*q* Model

Paalman–Pings^55^ corrections were applied, and the effect evaluated. A longer discussion on this is found in the Supporting Information, but it was mainly found that Paalman–Pings corrections have a positive, but small impact on the results. Therefore, our results include such a correction. A discussion on which model to be used is found in the Supporting Information, which concluded the “jump-diffusion” model to best find COM diffusion, while fitting each q individually (“per-*q*” model) was best for achieving comparable EISF.

#### Separation of Rotational and Translational Diffusion

In our QENS experiment, the observable apparent diffusion coefficient *D* = *D*(*D*_*r*_, *D*_*t*_) consists of contributions from both the rotational *D*_*r*_ and translational *D*_*t*_ diffusion. In practice, the separation of *D*_*r*_ and *D*_*t*_ is carried out with assumptions as below,^16,19^
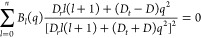
10and
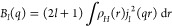
11where *j*_*l*_ represents the spherical Bessel function of the first kind and order *l*, and ρ_*H*_(*r*) denotes the radial hydrogen distribution function of the protein.

#### Relation between Diffusion, Molecular Mass, and Fractal Dimension

A relation between diffusion and molecular mass as well as fractal dimension (*d*_*F*_) has been proposed by Augé et al.,^56^
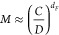
12which can be linearized to be
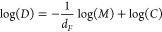
13

The constant *C* is dependent on the ”molecular family” considered, and needs to be parametrized. For this purpose, the α-synuclein diffusion measured by QENS (Fujiwara et al.^23^) was used together with the SAXS-data of α-synuclein (Ahmed et al.^57^). The fractal dimension of α-synuclein was computed from the SAXS data via linear fitting of log(*I*) vs log(*q*) at high *q*, using the limits of *q* = 2.0 Å^–^^1^ and *q* = 2.8 Å^–^^1^, as described in Johansen et al.^58^ The constant *C* was found to be 10 353. For the prediction of diffusion for Hst5, the SAXS data of Cragnell et al.^36^ were used.

### 2.4 Simulation Details

Trajectories from simulations of single-chain Hst5 in water using the A99SBN-ILDN (“A-ILDN”) force field with TIP4P-D water model, the CHARMM36m (“C36m”) force field with TIP3P water model modified for CHARMM, and the CHARMM36IDPSFF (“C36IDPS”) force field with TIP3P water model modified for CHARMM, were obtained from Henriques et al.^43^ and Jephthah et al.,^59^ and processed as described below. The simulations in this study were performed using the GROMACS software^60−64^ version 2019.2, with Amber ff99SB-*disp* (“A-Disp”) together with the TIP4P-derived water model a99SB-disp force field and the accompanying parameters for ions.^65^ These force fields were chosen since they have all previously been shown to accurately describe the structural properties of Hst5.^43,59,66^ The choice of A-Disp for the crowded simulations was motivated by it being a ”balanced” force field, intended to work for both globular and disordered proteins, which may be of importance in crowding studies as a possible outcome of crowding of IDPs is the induced ordering of the protein.^67^ The leapfrog integrator was used with a time step of 2 fs to compute the equations of motion. The LINCS algorithm was used to constrain bonds involving hydrogen atoms.^68^ A 12 Å cutoff was used for short-range electrostatic and Lennard-Jones interactions. For long-ranged electrostatic interactions, Particle-Mesh Ewald was used with a fourth order interpolation and 1.6 Å grid spacing.^69^ A Verlet neighbor list, updated every 100 fs was used, with a cutoff of 10 Å. Long-range dispersion corrections were applied to energy and pressure. Separate temperature baths with a velocity-rescale thermostat were coupled to the protein and solvent including ions at a temperature of 300 K and a relaxation constant of 0.1 ps.^70^ A Parrinello–Rahman barostat was applied, with the pressure fixed to 1 bar and setting relaxation time to 2 ps, whereas the isothermal compressibility was 4.5 × 10^–5^ bar^–1^. Periodic boundary conditions were applied in all directions. For the crowded simulations, a box with an initial side length of 9.4 nm was used (a box geometry was also used in the case of single-chain simulation) to insert the adequate amount of protein chains, then the box size was increased to 10 nm, to attain the correct protein concentration (two chains in case of 10 mg/mL protein concentration, ten chains in case of 50 mg/mL protein), and thereafter solvated with standard GROMACS tools. The increase in box size was to ensure a minimum distance between the box and the proteins. Sodium and chloride ions were inserted to both neutralize the charge of the proteins and to achieve a salt concentration of 150 mM. The final system sizes were 37 628 solvent molecules, 111 sodium ions, and 116 chloride ions for single-chain simulation, 30 389 solvent molecules, 90 sodium ions, and 100 chloride ions for a protein concentration of 10 mg/mL and 29 282 solvent molecules, 90 sodium ions, and 140 chloride ions for a protein concentration of 50 mg/mL. All chains had a starting conformation as a linear molecule, built in PyMol version 1.8 (Schrödinger, LLC). Initial energy minimization was performed with the steepest descent algorithm, followed by a stability equilibration run for 2 ns in the canonical ensemble (*NVT*: constant number of particles, volume and temperature). Thereafter a pressure stabilization was performed in the isobaric–isothermal ensemble (*NPT*: constant number of particles, pressure and temperature) for 2 ns. Production simulations were performed for 1200, 4000, and 4700 ns for single-chain simulations, 10 mg/mL protein concentration simulation and 50 mg/mL protein concentration simulation, respectively, using five replicates thus a total of 6, 20, and 23.5 μs. Any other settings were left as default, as determined by the GROMACS software. For computing the viscosity of the pure solvent A-Disp, 7198 molecules of A-Disp water was added initially in a cubic box with a side length of 6 nm, resulting in an initial molecular density of 33 molecules/nm^3^. A simulation was performed as above, with the following deviations: Three replicates were used, the NPT pressure equilibration was performed for 10 ns, and the production run was 30 ns long. The correctness of this simulation was confirmed by calculating the diffusion of the A-Disp water via mean square displacement (see below), which was found to be 1.9 × 10^–5^ cm^2^/s - the same as was originally found by Robustelli et al.^65^ All simulation trajectories have been used in full, without removing any initial part of the trajectories. This may introduce a small bias from the choice of starting structure. The influence of such bias has been deemed negligible from the convergence assessment, which can be found in the Supporting Information. For the production of EISF from trajectories, the program MDANSE^71^ was used (for further details on the algorithm used in MDANSE, see the Supporting Information).

#### Cluster Analysis of Molecular Dynamics Simulations of Proteins at a Concentration of 50 mg/mL

The GROMACS tool mindist was used to compute the minimum *c*–α carbon distance between all the chains in the simulation box for each replicate. Two cut-offs for defining whether two chains are in a cluster were used, 6 and 7 Å, as these are in the range used in similar applications.^72,73^

#### Diffusion Parameters from Molecular Dynamics Simulations

Transnational diffusion was determined by calculating the mean square displacement, MSD (by GROMACS standard tools) using the Einstein relation MSD = 6*D*_*t*_t, where *D*_*t*_ is translational diffusion and t time. A straight line was fitted to the region of time = 0 and time = 4.6 ns, corresponding to the coherence time of IN16B (with linearity of the region being ensured by computing R^2^ for each fit). This was completed for all chains in each replicate, averaging across all replicates and chains. The calculated translational diffusion for all individual chains can be found in the Supporting Information Tables S4–S8 and S10. The periodic boundary conditions were treated to ensure that no molecules were broken or diffusing across the simulation box, ensuring a continuous trajectory.

To account for the finite box used in the simulation, the correction for translational diffusion by Yeh and Hummer^74^ was used. Previous simulations of Hst5 by Henriques et al. used a rhombic dodecahedron simulation box rather than a cubic box, so in this case the constant ξ for FCC-lattice computed by Hasimoto^75^ was used, with the side-length being the length of the side of a unit cell with the rhombic dodecahedron inscribed. As well for the simulation by Henriques et al., viscosity of the pure solvent was taken from von Bülow et al.^72^ as it used the TIP4P-D water model. The corresponding viscosity for the CHARMM modified TIP3P water model was taken from Ong and Liow.^76^ The diffusion constants obtained were also further corrected for the discrepancy of using H_2_O in the simulation while using D_2_O in the experiment, by multiplying the ratio of viscosity between H_2_O and D_2_O. The correction also requires the viscosity of the system simulated, which was computed via an Einstein relation,
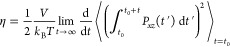
14as described by Hess.^77^ For single-chain simulations, the HYDROPRO software^78^ was also used for calculating translational diffusion, as an alternative. In this case, no corrections for the finite box-size is needed, as the structure is used directly. As well, when using HYDROPRO, the viscosity of D_2_O was used as input directly rather than correcting the H_2_O and D_2_O viscosity difference postprocess. For the computation of autocorrelation of the translational diffusion from HYDROPRO, the following definition of autocorrelation was used,
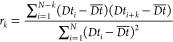
15where *Dt*_*i*_ is the translational diffusion at snapshot index *i*,  the mean translational diffusion, and *N* the total number of snapshots used.

## 3 Results and Discussion

### 3.1 Trends in Apparent COM Diffusion Coefficient

**Figure 1:**
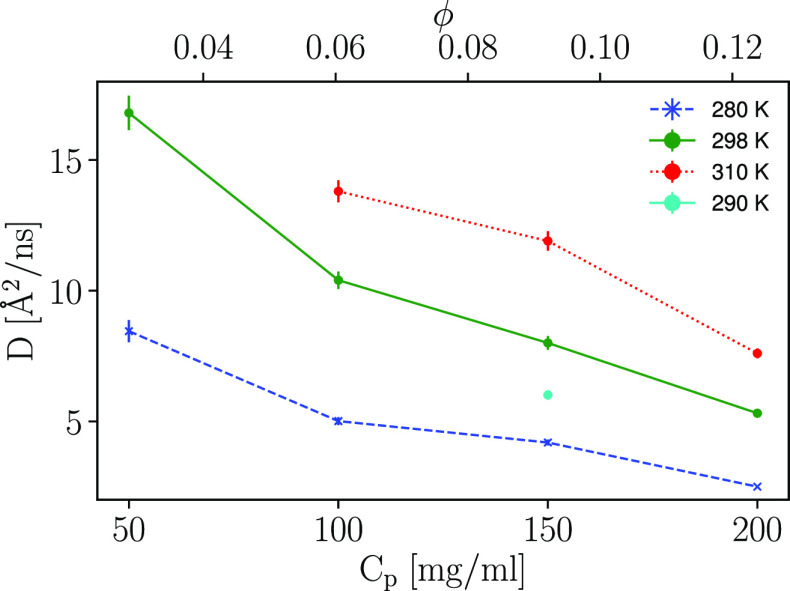
Apparent COM diffusion coefficient *D* versus the protein concentration *c*_*p*_ (lower *x*-axis) and protein volume fraction ϕ (upper *x*-axis). The symbols denote the experimental results obtained from fitting the IN16B QENS spectra in terms of Fickian diffusion (eq 2) for *D*, using jump-diffusion model for internal diffusion. Data shown is for a salt concentration of 150 mM NaCl, with the Paalman–Pings correction applied.

The apparent diffusion coefficient (*D*) of Hst5 was determined from the experimental QENS data using the jump-diffusion model with Paalman–Pings corrections applied (see discussion in the Supporting Information). The results are shown in Figure 1 as a function of both protein concentration and temperature. A consistent downward trend in apparent diffusion with increasing protein concentration is observed, indicating that crowding induces a reduction in the COM diffusion of Hst5. The temperature effect is, in relative terms, consistent across the different protein concentrations, increasing almost 3-fold from lowest to highest temperature. The data point for 50 mg/mL and 310 K is missing in the data set since we, for the IDP of the size used in this study, reached the limitations of technique at IN16B; i.e., the combined high speed-diffusion and comparably low protein concentrations did not provide feasible data. Considering the concentration dependence, the results indicate that the dynamics changes more drastically at lower protein concentrations. This is, however, not a consistent trend, as indicated by the small increase in slope between protein concentration 150–200 mg/mL, with the exception of the 298 K data, where the slope remains constant in the interval 100–200 mg/mL.

QENS experiments give the apparent diffusion *D*, which convolutes translational *D*_*t*_ and rotational diffusion *D*_*r*_. For globular proteins, Roosen-Runge et al.^19^ have demonstrated a procedure to deconvolute these two contributions. This was achieved by modeling their globular protein as an ellipsoid, using Perrin factors to achieve the dilute limit of rotational- and translational diffusion, as well as using a relation of how rotational diffusion changes with increasing crowding. The latter is valid for charged spheres (cf. eq 10); hence, it is not certain that this methodology is valid for IDPs, both considering the deconvolution itself, and the rotational diffusion relation. Additionally, Fagerberg et al.^37^ have shown that aggregation may take place at protein concentrations larger than 50 mg/mL. Therefore, this procedure is not applied.

**Figure 2:**
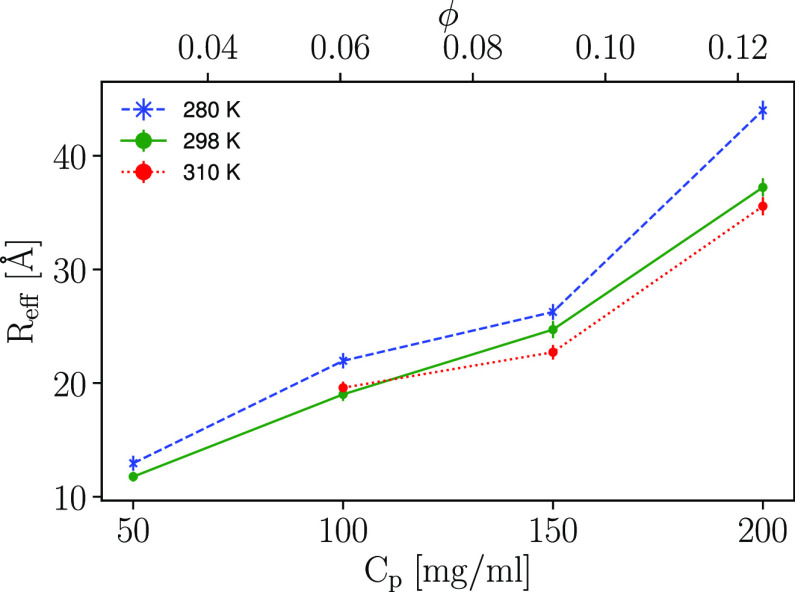
“Effective” radius of hydration *R*_e*ff*_ obtained using the Stokes–Einstein relation (eq 9) from the experimental diffusion coefficients to assess if the temperature dependence of the results is a consequence of changes in solvent properties solely. The diffusion coefficients used are those displayed in Figure 1 (150 mM NaCl concentration). The radius obtained represents an “effective” radius or “pseudo-hydrodynamic” radius since the observable apparent diffusion *D* is an implicit function of both the rotational and the translational diffusion. Error bars may be smaller than the symbol size.

As one may expect from the equipartition theorem, a higher temperature results in a faster diffusion. The temperature dependence on solvent properties may also be a factor in regard to the temperature dependence of the observed apparent diffusion. To evaluate this further, eq 9 is applied, however, this equation assumes translational diffusion, yielding the hydrodynamics radius (*R*_*h*_). Instead, an “effective” radius (*R*_*eff*_) is considered here. The important observation made from applying eq 9 is whether the temperature dependence of the observed diffusion is a consequence of changes in solvent properties, which would yield a similar *R*_*eff*_ across temperatures, or if additional explanations are necessary. Results are shown in Figure 2.

At low protein concentration, the *R*_*eff*_ differs only slightly between the different temperatures, 12.9 and 11.8 Å, at 280 and 298 K, respectively (a relative difference of 10%), indicating that the temperature dependence is Stokesian. At higher protein concentrations, the picture is again blurred by the possibility of aggregation, but the determined *R*_*eff*_ displays a difference of 15% at 100 mg/mL, 5% at 150 mg/mL, and 20% at 200 mg/mL.

Another step further would be to use *R*_*eff*_ to determine an estimate for the radius of gyration (*R*_*g*_). Employing the relation between *R*_*g*_ and *R*_*h*_ as suggested by Nygaard et al.,^79^ and assuming *R*_*h*_ = *R*_*eff*_, it is found that *R*_*g*_ is 8.1 and 6.0 Å at 280 and 298 K, respectively, at 50 mg/mL protein concentration. This is far from the value of 12.4 Å measured by Fagerberg et al.^37^ at the same protein concentration, indicating that all approximations assumed are not valid.

### 3.2 Effect of Salt Concentration

**Figure 3:**
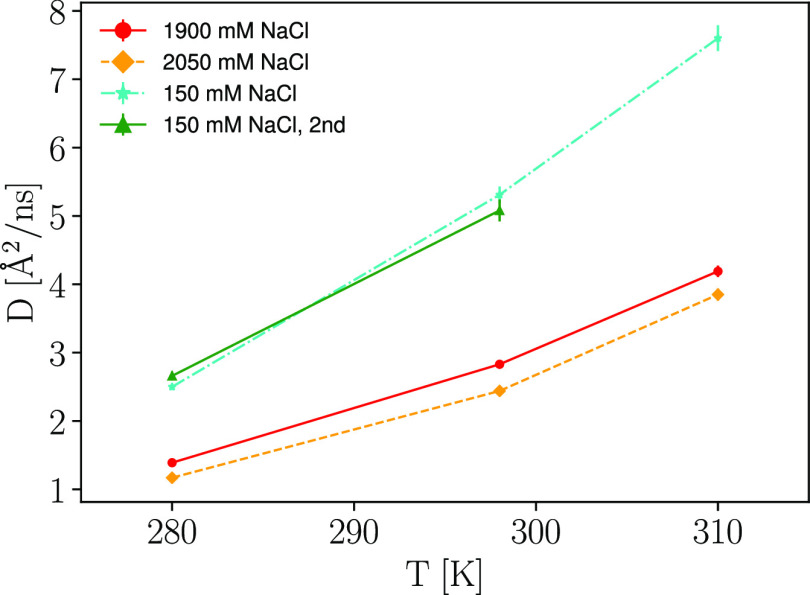
Apparent COM diffusion coefficient *D* obtained from the IN16B QENS data, subsequent to Paalman–Pings corrections, by fitting eq 1 and therein imposing eqs 2 and 3 for samples with a protein concentration of 200 mg/mL, while varying the amount of salt and temperature *T*.

At a protein concentration of 200 mg/mL, the effect of different salt concentrations were considered. Two different procedures were used regarding sample preparation, with or without dialysis, which produces different salt concentrations in the solution (a discussion and estimation of these can be found in Supporting Information). Furthermore, in order to verify the reproducibility, and considering the limited access to experimental beamtime, a single sample was measured twice with several months in-between the measurements. The apparent COM diffusion coefficient obtained by these measurements are shown in Figure 3.

There is a clear downshift in diffusion as the salt concentration increases. The relative temperature effect seems unaffected by the level of salt, with an increase in diffusion by approximately a factor of 3 in the temperature range between 280 to 310 K.

An increase in salt concentration alters the solvent properties. Of importance is the viscosity changes, which can be determined by the equations of Goldsack and Franchetto,^80,81^ which are valid in the salt concentration range considered. Calculations can be found in Supporting Information. Using these viscosities, *R*_*eff*_ was determined via the Stokes–Einstein equation (eq 9), which can be used to assess the effect on solvent properties by increases in salt. A considerable difference for the different levels of salt is still found (see Figure S9), suggesting that a large amount of salt has additional effects than changing solvent viscosity. From literature, slowdown of diffusion with increasing salt concentration has also been observed in QENS measurements of BSA and YCl_3_.^82^ In that case, the results were explained by salt-induced clustering, which might also be an explanation here. However, it should be emphasized that the salt used in the aforementioned study was trivalent and the protein studied was globular, which is significantly different compared with the current case. Another possible explanation for the decrease in diffusion with increasing salt concentration can be found by considering the coarse-grained simulations at different salt concentrations previously performed by Fagerberg et al.^37^ In these simulations, a larger *R*_*g*_ was found at low salt conditions, speculatively due the stronger charge-repulsion found at low salt concentrations as Hst5 has a fairly high net charge. Considering Hst5 to be an ellipsoid, a more extended structure would mean the polar semiaxis would be longer, and the equatorial semiaxis would be smaller. Applying the equation for diffusion of an ellipsoid moving randomly, reported by Berg,^83^ this would mean an increase in diffusion. An increase in diffusion at low salt concentration would be equivalent with a decrease in diffusion at high salt concentration, in line with the results shown here. The difference in salt for the samples was achieved not by adding salt, but by abstaining from dialyzing the samples intended to have high salt concentration. This, however, also reveals the importance of sample preparation. The lack of proper sample preparation may introduce excessive amounts of salt, yielding lower than expected diffusion rates.

### 3.3 Comparison with Simple Geometries

To appreciate the deviation of Hst5 from the diffusive behavior of more simple geometries, we here determine the corresponding effective diffusion for a sphere and an ellipsoid (Table 1). For both geometries, the translational diffusion is obtained through the Stokes–Einstein equation, and the rotational diffusion through Einstein–Smoluchowski relation, with the difference being the friction factor. Using the *P*(*r*) from SAXS as found by Cragnell et al.,^36^ and the *R*_*g*_ found by the same SAXS-data (with *R*_*h*_ estimated from the relation by Nygaard et al.), the apparent (or effective) diffusion is found to be 1.96 Å^2^/ns—a magnitude off (!).

**Table 1:** Computed Diffusion Using Simple Geometrical Models^a^

model	*D*_*t*_	*D*_*r*_	*D*
sphere	1.35	0.005	1.96
ellipsoid	1.56	0.1	2.66

a Translational diffusion *D*_*t*_ and apparent diffusion *D* in units of Å^2^/ns and rotational diffusion *D*_*r*_ in units of 1/ns. For the spherical case, the relation of Nygaard *et al.* was used to compute *R*_*h*_ from *R*_*g*_.

A better approximation of the shape can be found by fitting an ellipsoid to the SAXS-data. Such fitting yields polar semiaxis *a* = 32.9 Å, and equatorial semiaxes *b* = 5 Å, respectively. Perrin-factors are then used to attain translational- and rotational diffusion, following Roosen-Runge et al.^19^ Again, using P(r) as found by SAXS-measurement, the apparent diffusion becomes 2.66 Å^2^/ns - still significantly smaller than the experimentally determined value.

On a related note, it is possible to compute *R*_*h*_ via Perrin-factors, offering an alternative to the relation by Nygaard et al. Inspecting the numbers determined, 14.61 Å (Nygaard-relation) and 12.65 Å (Perrin-factors), it is observed that if the Perrin-factors are used, a *R*_*h*_ smaller than *R*_*g*_ is found. Furthermore, using the *R*_*h*_ obtained through Perrin-factors, the apparent diffusion becomes smaller. This would make a case against using Perrin-factors to calculate *R*_*h*_ in this case, which is not unexpected, since the relation by Nygaard et al. was specifically developed for IDPs and thus should indeed perform better for Hst5.

#### 3.3.1 Scaling Laws for Colloidal Hard Sphere Suspensions

**Figure 4:**
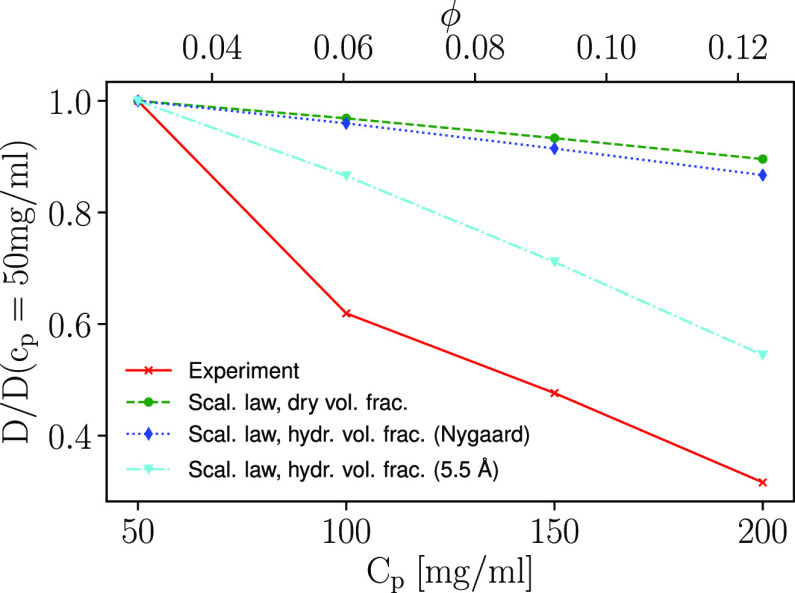
Comparison of diffusion constants obtained by the colloidal scaling law of Banchio and Nägele (eq 16), utilizing the deconvolution procedure by Roosen-Runge et al. (eq 10), and the experimental data. Experimental data is shown for 150 mM NaCl, at 298 K, with Paalman–Pings corrections used. The scaling law is combined with different assumptions on *R*_*h*_, as it assumes a volume fraction based on effective *R*_*h*_.

Several scaling laws for diffusion constants with increasing crowding have been published, each making different assumptions on particle properties.^24,84,85^ Here, as both translational- and rotational diffusion relationships were derived, and since Hst5 is a charged peptide, we choose the scaling law that assumes charged spheres by Banchio and Nägele,^24^
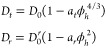
16with *a*_*t*_ = 2.5 and *a*_*r*_ = 1.3 and the effective hydrodynamic volume fraction ϕ_*h*_. This scaling law has successfully described the change in diffusion for BSA protein.^19^ We stress that the applicability of such scaling models based on spherical colloids may be severely limited to describe strongly nonspherical proteins.^86^ Using the scaling law in combination with the implicit function *D* = *D*(*D*_*r*_, *D*_*t*_) by Roosen-Runge et al., eq 10, to calculate the observable *D*, would in this case be valid as the scaling law of Banchio and Nägele assumes charged spheres, thus circumventing the uncertainty of whether the deconvolution procedure is valid for IDPs. A comparison can thereafter be achieved by dividing with the value attained at 50 mg/mL, in both the colloid model case and the experimental case (see Figure 4). It is noted that ϕ_*h*_ in eq 16 differs from the experimental as-prepared protein volume fraction ϕ (eq 4) (“dry volume fraction”) because the COM diffusion is governed by *R*_*eff*_ that includes a hydration shell.^19^ In our QENS experiment, the difference in scattering cross section between hydrogen and deuterium makes it possible to probe the (nondeuterated) protein exclusively, i.e., no hydration shell is considered in the assumptions of the relative amplitudes β and  of the contributions to the scattering signal (eq 1), and any effect from a change in the solvent dynamics itself near the protein surface is neglected.^87^ We here assume *ϕ*_h_ = (*R*_*h*_/*R*_*g*_)^3^ϕ to approximately accommodate this difference. *R*_*h*_ was approximated by the relation by Nygaard et al., as before, however, we also consider that this relation is one of many suggested, and that this has a fairly low *R*_*h*_/*R*_*g*_ ratio (1.06) for Hst5. Nygaard et al. also points out that for an idealized sphere, *R*_*h*_/*R*_*g*_ = 1.28, but an even larger ratio is found by using *R*_*h*_ = *R*_*g*_ + hydration shell, and by assuming a hydration shell of 5.5 Å, using data from Perticaroli et al.,^87^ who found the perturbed number of water molecules near a globular protein corresponds to a hydration layer smaller than 5.5 Å. This gives a ratio of 1.40. Results from using both ”dry volume fraction” and ”hydrodynamic volume fraction”, comparing the scaling law with our measurements, are shown in Figure 4.

Using the relation by Nygaard et al., and accounting for the difference in hydrodynamic/dry volume fraction, only returns a small difference. An increased *R*_*h*_ seemingly also gives better agreement with the experimental result, but not even the worst-case scenario considered here can fully account for the low diffusion rate. In this context, previous research has indicated that hydration of IDPs may be different compared to that of globular proteins.^88^

#### 3.3.2 Prediction Using Fractal Dimension as a Variable

Instead of approximating Hst5 as a sphere or an ellipsoid, an analytical expression for diffusion using fractal dimension and molecular mass as input variable has been suggested by Augé et al.,^56^ as can be viewed in eq 12. After parametrization of this relation using QENS data of α-synuclein, a prediction of diffusion for Hst5 of 41 Å^2^/ns is obtained. This is more than double the experimentally determined value of about 17 Å^2^/ns at the lowest measured protein concentration. To consider the crowding effect, it is observed from QENS that increasing protein concentration from 50 to 100 mg/mL decreases the diffusion by 40%. Such a crowding response would still not be enough to validate the model. Of note in this context is the sensitivity of the prediction—a difference of 0.05 in fractal dimension can yield a difference in diffusion of almost 10 Å^2^/ns, which is of relevance since the region from which the fractal dimension is calculated is somewhat noisy and prone to the accuracy of buffer subtraction.

### 3.4 Molecular Dynamics Simulations

Previously, atomistic simulations of single-chain Hst5 have been performed by Henriques et al.,^43^ using the Amber99SBN-ILDN force field (shortened “A-ILDN” here), and Jephthah et al.^59^ using the CHARMM36m (“C36m” for short) and CHARMM36IDPSFF (“C36IDPS” for short) force fields. Structural data obtained from all of these force fields have previously shown to be in agreement with experimental SAXS data of Hst5, thus showing their suitability. We also perform a simulation with the Amber99SB-disp (“A-Disp” for short), which also has been used to predict structural dimensions of Hst5. The determined translational diffusion can be found in Table 2.

**Table 2:** Computed Translational Diffusion Coefficients (*D*_*t*_) Obtained Using Different Force Fields and Different Methods^a^

force field	*D*_*t*_	*D*_*t*_ incl. crowding	HYDROPRO prediction of *D*_*t*_
A-ILDN	22.4 ± 0.3	21.9 ± 0.3	13.2 ± 0.8
A-Disp	16.0 ± 0.2	15.7 ± 0.1	13.4 ± 0.8
C36m	55.6 ± 1.3	54.3 ± 1.2	13.2 ± 0.9
C36IDPS	59.8 ± 0.9	58.4 ± 0.9	13.9 ± 0.9

a The “crowding” included is by using eq 16. The ± sign indicates standard deviation. All units in Å^2^/ns.

Comparing the different numbers obtained, there is a clear difference between using a CHARMM-based force field with TIP3P based water model, or an Amber-based force field with a TIP4P-D based water model, with the former predicting much faster translational diffusion. The translational diffusion produced from these simulations is not the same as the effective diffusion found in QENS, but can still be used for comparison with our experimental results, at least as a lower bound. The corresponding diffusion coefficient found by QENS is 16.8 ± 0.66 Å^2^/ns, at a concentration of 50 mg/mL. Therefore, it is seen that most force fields overestimate the translational diffusion, with the exception of A-Disp, which, taking standard deviation into account, is barely in agreement with experiment.

No concentration dependence on the structural properties of Hst5 has been observed, between low protein (≈ 6 mg/mL) and 50 mg/mL protein concentration experimentally,^37^ but the same may not be true for dynamical properties. Therefore, the difference in diffusion may be attributed to the differing protein concentration in the simulation, which here uses infinite dilution, and the compared experiment, which here is at a protein concentration of 50 mg/mL. eq 16 can provide an estimate, given that it assumes charged hard spheres, to evaluate this concern. Using the diffusion constants obtained from the infinite-dilution simulation and these scaling laws, a recalculated translational diffusion is obtained, see Table 2. It is mainly observed that the change is small, only qualitatively showing that the A-Disp force field is slightly underestimating the diffusion, rather than being within experimental error.

**Figure 5:**
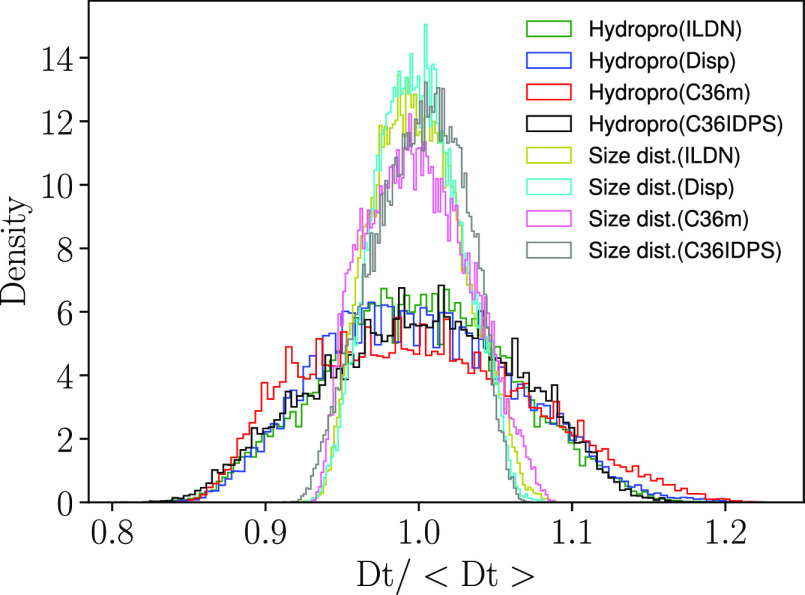
Reduced distribution of translational diffusion computed for each snapshot using HYDROPRO or *R*_*g*_, combined with the relation of Nygaard et al. to obtain *R*_*h*_, and the Stokes–Einstein relation. The ”reduction” is done by dividing with the mean translational diffusion for each case.

As an alternative procedure, translational diffusion can be computed via the HYDROPRO program.^78^ Results from this procedure are found in Table 2. As the translational diffusion is somewhat smaller than the effective diffusion found by QENS, this estimate is surprisingly close to experiment, given that HYDROPRO was parametrized with crystal structures (while Hst5 is an IDP). However, adding the fact that experiment was performed at a higher protein concentration, thus subject to a crowding effect, may suggest the calculated number is an underestimation. Using the HYDROPRO approach, the translational diffusion was calculated for each snapshot, yielding a distribution of diffusion, see Figure 5.

**Figure 6:**
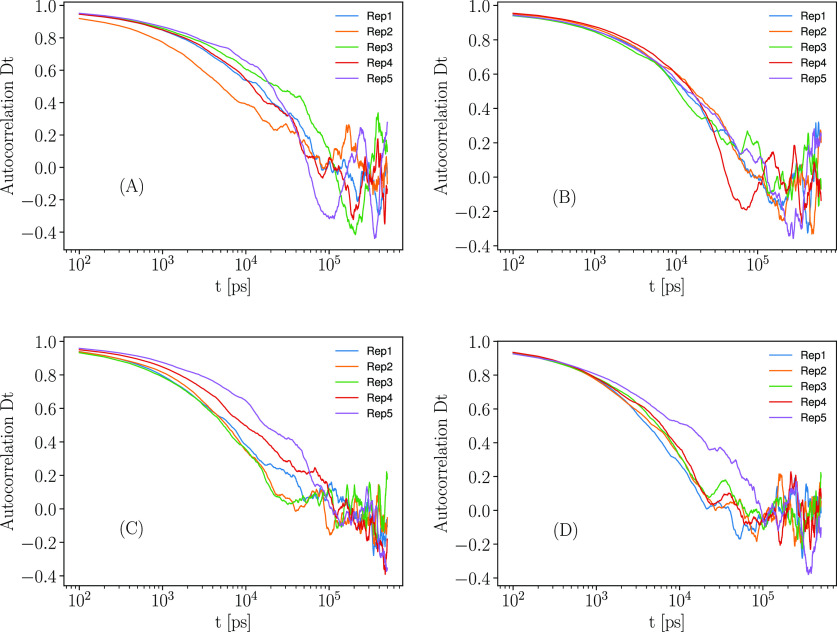
Computed autocorrelation of the translational diffusion (eq 15) for all the replicates using the A-ILDN force field (A), the A-Disp force field (B), the C36m force field (C), and the C36IDP force field (D), using the HYDROPRO software. The resolution here is one data point every 100 ps.

To show that the “HYDROPRO-approach” is not a simplistic mirroring of the size distribution of particles (defined in terms of *R*_*g*_), *R*_*g*_ was determined for each individual snapshot, from which the translational diffusion was calculated via Stokes–Einstein equation. As we know that this procedure yields lower average translational diffusion, both distributions were divided by the average translational diffusion in Figure 5. As expected HYDROPRO produces a broader distribution, indicating that the particle shape heterogeneity, and not just the particle size heterogeneity, is a contributing factor. As well as considering distributions of translational diffusion, the HYDROPRO approach also allows for the computation of the autocorrelation of the translational diffusion (eq 15), achieved for each individual chain for all force fields in Figure 6.

The autocorrelation time is much longer than the time observed in the QENS experiment, regardless of the force field used, which based on an energy resolution of 0.9 μeV full width at half maximum (fwhm) would not be longer than a few nanoseconds. This implies that the QENS experiment yields ensemble averages over the system, and not an average over time.

#### 3.4.1 Elastic Incoherent Structure Factors

**Figure 7:**
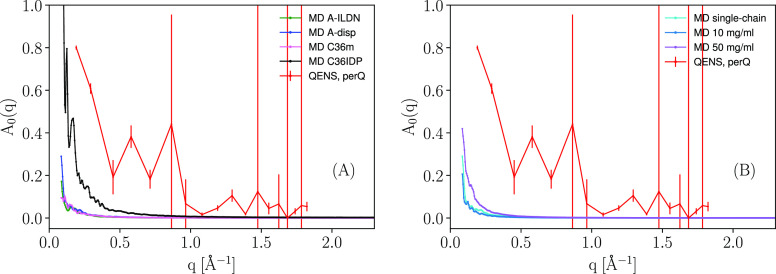
Comparison of the elastic incoherent structure factor (EISF) *A*_0_(*q*) between experiment (eq 1, protein concentration *c*_*p*_ = 50 mg/mL, 150 mM salt, temperature *T* = 298 K, not imposing the *q*-dependence in the model) and simulation. (A) Comparing the single-chain simulations with experiment. (B) Comparing the crowded simulations with experiment.

The Elastic Incoherent Structure Factor (EISF) was calculated from the simulation using the MDANSE software^71^ (details of how this computation is performed can be found in Supporting Information, section 6.), the results are shown in Figure 7.

Remarkably, the C36IDPS force field yields a perhaps somewhat closer agreement with experiment, despite its previously shown poor results in terms of diffusion. It is also seen to be fairly noisy, which may explain the superficial better agreement with experiment as a coincidence, when scaling the curve as to have *A*_0_(0) = 1. We speculate that the rather featureless EISF, obtained by both experiment and simulation, corroborates the effective average over the fluctuating shape of the protein. The single drop with increasing *q* in this picture reflects an effective diffusive mean-free path within the protein. The corresponding experimental EISF at higher protein concentrations is found in the Supporting Information, where there is indication of the apparent mean free path decreases with increasing crowding, though the fairly large error bars partly obscures this view. A difference in EISF, can in this case, be caused by the different conditions of the experiment and the simulation. In terms of *q*, the simulation has its limits set by the simulation box size and the precision of the coordinates. For the simulation of Henriques et al., which used a rhombic dodecahedron as box geometry, this amounts to *q*-values of about 0.05–600 Å^–1^. Similar values apply for the crowded simulations, which used a cubic box geometry. The QENS measurements on the other hand are restricted to *q*-values of 0.19–1.8 Å^–1^. In terms of energy resolution, the experimental resolution of 0.9 μeV fwhm result in that motions slower than a few nanoseconds are perceived as immobile in the experiment. Correspondingly, the finite length of the simulated trajectory will render slow motions beyond such cutoff invisible in the simulations. On the other end, very fast motions may not be captured due to the finite sampling in the simulations.

#### 3.4.2 Effect of Crowding

Starting with structural features from the simulations, *R*_*g*_ was found to be 12.2 Å in the case of 10 mg/mL protein concentration and 12.9 Å for the 50 mg/mL protein concentration simulation, which should be compared with the single-chain simulation, which produced a *R*_*g*_ of 13.1 Å (all numbers for A-Disp force field, having a standard deviation of 2.2 Å). The commonly accepted experimental value at low protein concentration (approximated as infinite dilution) is 13.8 Å,^36^ though other experimentalists have found values as low as 12.4 Å.^37^ This is however not the first time Hst5 has been simulated with the A-Disp force field: Jephthah et al.^59^ found *R*_*g*_ of 12.9 Å. Shrestha et al.^66^ on the other hand, found a *R*_*g*_ slightly below 12 Å with standard simulation methods and slightly above 12 Å with enhanced sampling, though showing good agreement with SAXS data despite the somewhat small values of *R*_*g*_. Hence, considering the variation between different experiments and simulations, it would seem that crowding in these simulation does not induce a change in *R*_*g*_. As can be gathered from the last snapshots of the simulations (Figures S18), irreversible aggregation does not seem to have occurred for the protein concentration considered. Both of these observations are in line with previous crowding experiments.^37^ Considering secondary structure in terms of the phi/psi dihedral angles, we first note that the Ramachandran plot for the single-chain simulation using the A-Disp force field (Figures S23, left) is similar to the one produced by Jephthah et al.^59^ Second, by comparing the Ramachandran plots for the different protein concentrations (Figure S23), it is observed that they are practically indistinguishable. This would further indicate that, structurally, there is no change upon increasing concentration in the concentration span investigated.

##### Clustering Analysis

An analysis was performed to investigate the possible formation of transient clusters. As can be seen from Figures S19–S21, the proteins are very active in forming and breaking clusters of varying size. The exact numbers for these depends on how a cluster is defined, but using a metric found in similar studies with globular proteins^72,73^ and testing two different cut-offs, it is found that on average, there are two clusters present, with a total of six protein chains participating, and the largest cluster being three to four protein chains (Table S9). This analysis may depend on the size of the box used (which determines the number of protein chains in the system, keeping the concentration constant).

##### Diffusion

**Figure 8:**
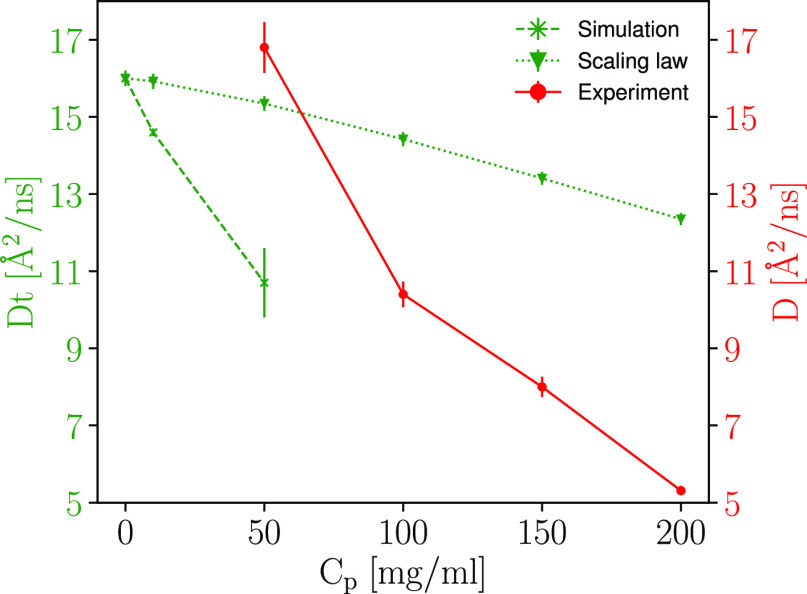
*D*_*t*_ as obtained from simulation of Hst5 at different crowding conditions with the A-Disp force field, the scaling law using only the translational diffusion part – no rotational part, and the experimental data. The reader is reminded that the experimental data is an apparent diffusion (*D* = *D*(*D*_*t*_, *D*_*r*_)).

Regarding the diffusion, there is a clear decrease in *D*_*t*_ with increasing crowding. Using the A-Disp force field, infinite dilution showed *D*_*t*_ of 16.0 (standard deviation 0.2) Å^2^/ns (as discussed above), while crowded simulations predicted *D*_*t*_ of 14.6 (standard deviation 0.1) and 10.7 (standard deviation 0.9) Å^2^/ns for 10 and 50 mg/mL protein concentration, respectively. Even if, for any individual chain, fluctuations may be large, this still points to a crowding effect greater than the crowding scaling law of choice indicated. The data is shown in Figure 8, together with the *D*_*t*_ part of the scaling law (eq 16) and the experimental data. It is observed that the scaling law, used with the Nygaard relation here, also for the simulation data underestimates the effect of crowding. In this case, the difference is not due to irreversible aggregation, which might have been postulated in the experimental case.

However, the presence of transient clusters may contribute to the decrease in diffusion. Comparison of the diffusion on a per-replicate basis with the different metrics of transient clustering (Figure S22), there is an indication that the diffusion is dependent on the total number of proteins participating in transient clusters. This is also shown by making a linear regression of the data and computing *R*^2^, see Table S11. It should be stressed that this is only an indication, given the few data points available here.

Comparing with the experimental value of 16.8 ± 0.66 Å^2^/ns (*D*) at 50 mg/mL with the predicted value 10.7 Å^2^/ns (*D*_*t*_) from the simulation of 50 mg/mL protein concentration, it might be hard to conclude whether the model is in line with experiment or not—as a lower bound, any value below the experimentally found value could be argued reasonable. To achieve a more fair comparison, we studied the relative ratios between *D* and *D*_*t*_ as found by Fujiwara et al.,^23^ where the ratio was found to be 1.27 (while the same ratio using the ellipsoid approximation as shown above is 1.7). With this ratio, an approximate *D* of 13.6 Å^2^/ns is found, indicating simulation to yield slower dynamics than experimental evidence. The ratio may differ between different systems, but as a first approximation (and a better approximation than the ellipsoid approximation, as Fujiwara et al. studied an IDP), the model used seem to underestimate the dynamics of Hst5. Although the system is crowded with protein to a volume fraction of 0.03, the water model may indeed play a role in this underestimation, given its deviation from experiment.

Wang et al.^89^ has previously measured diffusion of a globular and a disordered protein using NMR together with different crowders, finding a significant change in diffusion for both kinds of systems, though at more crowded conditions than studied here (300 mg/mL). However, pointing to other studies, Wang et al. notes that this occurs without a change in structure, which would be in line with the results from our simulations, but also partly in line with a study by König et al.^15^ When investigating the IDP prothymosin α, in the crowded conditions of eukaryotic cells, no change in chain dimensions was found but a slowdown of translational diffusion (factor of 1.5), though also finding chain compaction upon further crowding (induced by hyperosmotic stress).

In absolute numbers, our results have similar magnitude as those of Fujiwara et al., who showed α-synuclein to have *D* in the range of 9–16 Å^2^/ns for different temperatures at a protein concentration of 10 mg/mL. Two key differences should be mentioned in this context: The measurements of Fujiwara et al. were performed at a protein concentration of about 10 mg/mL, and α-synuclein is a much larger protein (14.5 kDa, while Hst5 is 3.0 kDa). Other QENS measurements performed with globular proteins show a magnitudal difference in *D*, as shown in Table 3. The difference in size should of course be considered here, as the molar mass range in the above studies is 57–150 kDa. However, BSA, which has slightly less than half the size of immunoglobulin, has similar diffusivity at dilute conditions, clearly indicating that larger molecular mass does not directly result in slower diffusion, since the shape also matters. This would in turn indicate that the magnitude faster diffusion found in the IDPs surveyed is not a simple consequence of their smaller molecular mass.

**Table 3:** Other Studies of Proteins Using QENS, Considering the Size of the Protein and the Obtained Apparent Diffusion

protein	size [kDa]	app. diff. [Å^2^/ns]	note
Hst5	3.0	2.5–17	this work
α-synuclein^23^	14.5	9–16	
immunoglobulin^20^	150	1–4	temperature of 293 K
BSA^19^	66.4	0–4	temperature of 280 K
GroEl^18^	57	0.5–1.8	temperature of 297 K

## 4 Conclusions

The apparent diffusion of the IDP Hst5 has been obtained by QENS under self-crowded conditions. A decrease in diffusion with increasing crowding is found, exceeding the decrease calculated from an established scaling law, which has previously been shown to describe the diffusion of globular proteins. We hypothesize that this strong decrease is a consequence of a minor degree of aggregation at higher crowding levels. The temperature dependence of the results is largely explained by the Stokes–Einstein equation. Increasing the salt concentration decreases the diffusion, a relation not described by changes in solvent properties (i.e., salt-dependent viscosity). Usage of simple geometries grossly underestimates diffusion constants, and analyzing structures from MD simulations with HYDROPRO indicate that the distribution of the diffusion not only depends on size. Crowded MD simulations also show a clear decrease of the diffusion constant with increasing crowding, in semiquantitative agreement with experiment. There is some uncertainty in this comparison, as the experimental observable is not directly comparable with the translational diffusion obtained from simulation, though a few suggestions on this relationship can be found in the literature. The EISF found in the experiment has too large error bars to be reasonably compared with the ones obtained from the simulation results. Under crowded conditions, the diffusion of the IDP Hst5 is more greatly influenced than the structural properties.

## Supporting Information Available

List of all samples measured with QENS, examples of QENS-spectra and fittings of these, EISF for different protein concentrations, discussion of fitting considerations (including figures of goodness-of-fit, L1 loss function, and L2 loss function), description of how salt content was computed when samples was prepared with dialysis, the equation of Goldsack and Franchetto, accompanied by a short description, a list of the computed viscosities for samples with excessive salt, a figure with computed effective radius for the different salt conditions, a figure and a table for the parameters obtained from the fitting of the “jump-diffusion”-model to the QENS data, and figures and data (including convergence information, simulation snapshots, computed translational diffusion for each chain, Ramachandran plots, cluster-analysis) for all simulations performed (PDF)
Movie of 90 ns of a 50 mg/mL Hst5 trajectory (MPG)

## Supplementary Material

jp1c08976_si_001.pdf

jp1c08976_si_002.mpg

## References

[ref1] Dyson H. J.; Wright P. E. Intrinsically unstructured proteins and their functions. Nat. Rev. Mol. Cell Biol. 2005, 6, 197–208. 10.1038/nrm1589. 15738986

[ref2] Radivojac P.; Obradovic Z.; Smith D. K.; Zhu G.; Vucetic S.; Brown C. J.; Lawson J. D.; Dunker A. K. Protein flexibility and intrinsic disorder. Protein Sci. 2004, 13, 71–80. 10.1110/ps.03128904. 14691223 PMC2286519

[ref3] Uversky V. N. Intrinsically disordered proteins and their “mysterious”(meta) physics. Frontiers in Physics 2019, 7, 10 10.3389/fphy.2019.00010.

[ref4] Moses D.; Yu F.; Ginell G. M.; Shamoon N. M.; Koenig P. S.; Holehouse A. S.; Sukenik S. Revealing the hidden sensitivity of intrinsically disordered proteins to their chemical environment. J. Phys. Chem. Lett. 2020, 11, 10131–10136. 10.1021/acs.jpclett.0c02822. 33191750 PMC8092420

[ref5] Wicky B. I.; Shammas S. L.; Clarke J. Affinity of IDPs to their targets is modulated by ion-specific changes in kinetics and residual structure. Proc. Natl. Acad. Sci. U. S. A. 2017, 114, 9882–9887. 10.1073/pnas.1705105114. 28847960 PMC5604010

[ref6] Ellis R. Macromolecular crowding: obvious but underappreciated. Trends Biochem. Sci. 2001, 26, 597–604. 10.1016/S0968-0004(01)01938-7. 11590012

[ref7] Zimmerman S. B.; Trach S. O. Estimation of Macromolecule Concentrations and Excluded volume effects for the Cytoplasm of Escherichia coli. J. Mol. Biol. 1991, 222, 599–620. 10.1016/0022-2836(91)90499-V. 1748995

[ref8] Fonin A. V.; Darling A. L.; Kuznetsova I. M.; Turoverov K. K.; Uversky V. N. Intrinsically disordered proteins in crowded milieu: when chaos prevails within the cellular gumbo. Cell. Mol. Life Sci. 2018, 75, 3907–3929. 10.1007/s00018-018-2894-9. 30066087 PMC11105604

[ref9] Banks A.; Qin S.; Weiss K. L.; Stanley C. B.; Zhou H.-X. Intrinsically Disordered Protein Exhibits Both Compaction and Expansion under Macromolecular Crowding. Biophys. J. 2018, 114, 1067–1079. 10.1016/j.bpj.2018.01.011. 29539394 PMC5883552

[ref10] Brangwynne C. P.; Tompa P.; Pappu R. V. Polymer physics of intracellular phase transitions. Nat. Phys. 2015, 11, 899–904. 10.1038/nphys3532.

[ref11] Cino E. A.; Karttunen M.; Choy W. Effects of Molecular Crowding on the Dynamics of Intrinsically Disordered Proteins. PLoS One 2012, 7, e49876 10.1371/journal.pone.0049876. 23189168 PMC3506533

[ref12] Bugge K.; Brakti I.; Fernandes C. B.; Dreier J. E.; Lundsgaard J. E.; Olsen J. G.; Skriver K.; Kragelund B. B. Interactions by disorder–a matter of context. Frontiers in Molecular Biosciences 2020, 7, 110 10.3389/fmolb.2020.00110. 32613009 PMC7308724

[ref13] Salvi N.; Abyzov A.; Blackledge M. Multi-timescale dynamics in intrinsically disordered proteins from NMR relaxation and molecular simulation. J. Phys. Chem. Lett. 2016, 7, 2483–2489. 10.1021/acs.jpclett.6b00885. 27300592

[ref14] Wang Y.; Benton L. A.; Singh V.; Pielak G. J. Disordered protein diffusion under crowded conditions. J. Phys. Chem. Lett. 2012, 3, 2703–2706. 10.1021/jz3010915. 23185649 PMC3505085

[ref15] König I.; Soranno A.; Nettels D.; Schuler B. Impact of In-Cell and In-Vitro Crowding on the Conformations and Dynamics of an Intrinsically Disordered Protein. Angew. Chem. 2021, 133, 10819–10824. 10.1002/ange.202016804. 33587794

[ref16] Grimaldo M.; Roosen-Runge F.; Zhang F.; Schreiber F.; Seydel T. Dynamics of proteins in solution. Q. Rev. Biophys. 2019, 52, E7 10.1017/S0033583519000027.

[ref17] Fleischer G.; Fujara F. NMR as a generalized incoherent scattering experiment. In Solid-State NMR I Methods. NMR (Basic Principles and Progress); Blümich B., Eds.; Springer, Berlin, Heidelberg, 1994; Vol. 30, pp 159–207. 10.1007/978-3-642-78483-5_4

[ref18] Anunciado D. B.; Nyugen V. P.; Hurst G. B.; Doktycz M. J.; Urban V.; Langan P.; Mamontov E.; O’Neill H. In Vivo Protein Dynamics on the Nanometer Length Scale and Nanosecond Time Scale. J. Phys. Chem. Lett. 2017, 8, 1899–1904. 10.1021/acs.jpclett.7b00399. 28388043

[ref19] Roosen-Runge F.; Hennig M.; Zhang F.; Jacobs R. M. J.; Sztucki M.; Schober H.; Seydel T.; Schreiber F. Protein self-diffusion in crowded solutions. Proc. Natl. Acad. Sci. U. S. A 2011, 108, 11815–11820. 10.1073/pnas.1107287108. 21730176 PMC3142006

[ref20] Grimaldo M.; Lopez H.; Beck C.; Roosen-Runge F.; Moulin M.; Devos J. M.; Laux V.; Härtlein M.; Da Vela S.; Schweins R.; et al. Protein Short-Time Diffusion in a Naturally Crowded Environment. J. Phys. Chem. Lett. 2019, 10, 1709–1715. 10.1021/acs.jpclett.9b00345. 30897330

[ref21] Grimaldo M.; Roosen-Runge F.; Zhang F.; Seydel T.; Schreiber F. Diffusion and Dynamics of γ-Globulin in Crowded Aqueous Solutions. J. Phys. Chem. B 2014, 118, 7203–7209. 10.1021/jp504135z. 24871685

[ref22] Braun M. K.; Grimaldo M.; Roosen-Runge F.; Hoffmann I.; Czakkel O.; Sztucki M.; Zhang F.; Schreiber F.; Seydel T. Crowding-Controlled Cluster Size in Concentrated Aqueous Protein Solutions: Structure, Self- and Collective Diffusion. J. Phys. Chem. Lett. 2017, 8, 2590–2596. 10.1021/acs.jpclett.7b00658. 28525282

[ref23] Fujiwara S.; Araki K.; Matsuo T.; Yagi H.; Yamada T.; Shibata K.; Mochizuki H. Dynamical Behavior of Human α-Synuclein studied by Quasielastic Neutron Scattering. PloS One 2016, 11, e0151447 10.1371/journal.pone.0151447. 27097022 PMC4838215

[ref24] Banchio A. J.; Nägele G. Short-time transport properties in dense suspensions: From neutral to charge-stabilized colloidal spheres. J. Chem. Phys. 2008, 128, 104903 10.1063/1.2868773. 18345924

[ref25] Beck C.; Grimaldo M.; Roosen-Runge F.; Braun M. K.; Zhang F.; Schreiber F.; Seydel T. Nanosecond Tracer Diffusion as a Probe of the Solution Structure and Molecular Mobility of Protein Assemblies: The Case of Ovalbumin. J. Phys. Chem. B 2018, 122, 8343–8350. 10.1021/acs.jpcb.8b04349. 30106587

[ref26] Bée M. A physical insight into the elastic incoherent structure factor. Phys. B (Amsterdam, Neth.) 1992, 182, 323–336. 10.1016/0921-4526(92)90034-P.

[ref27] Perticaroli S.; Nickels J. D.; Ehlers G.; Mamontov E.; Sokolov A. P. Dynamics and rigidity in an intrinsically disordered protein, β-casein. J. Phys. Chem. B 2014, 118, 7317–7326. 10.1021/jp503788r. 24918971

[ref28] Stadler A. M.; Koza M. M.; Fitter J. Determination of conformational entropy of fully and partially folded conformations of holo-and apomyoglobin. J. Phys. Chem. B 2015, 119, 72–82. 10.1021/jp509732q. 25494533

[ref29] Gallat F.-X.; Laganowsky A.; Wood K.; Gabel F.; Van Eijck L.; Wuttke J.; Moulin M.; Härtlein M.; Eisenberg D.; Colletier J.-P.; et al. Dynamical coupling of intrinsically disordered proteins and their hydration water: comparison with folded soluble and membrane proteins. Biophysical journal 2012, 103, 129–136. 10.1016/j.bpj.2012.05.027. 22828339 PMC3388209

[ref30] Puri S.; Edgerton M. How Does It Kill?: Understanding the Candidacidal Mechanism of Salivary Histatin 5. Eukaryotic Cell 2014, 13, 958–964. 10.1128/EC.00095-14. 24951439 PMC4135785

[ref31] Ruissen A. L. A.; Groenink J.; Helmerhorst E. J.; Walgreen-Weterings E.; van’t Hof W.; Veerman E. C. I.; Nieuw Amerongen A. V. Effects of histatin 5 and derived peptides on Candida albicans. Biochem. J. 2001, 356, 361–368. 10.1042/bj3560361. 11368762 PMC1221846

[ref32] Bennick A. Interaction of Plant Polyphenols with Salivary Proteins. Crit. Rev. Oral Biol. Med. 2002, 13, 184–196. 10.1177/154411130201300208. 12097360

[ref33] Wróblewski K.; Muhandiram R.; Chakrabartty A.; Bennick A. The molecular interaction of human salivary histatins with polyphenolic compounds. Eur. J. Biochem. 2001, 268, 4384–4397. 10.1046/j.1432-1327.2001.02350.x. 11502198

[ref34] Melino S.; Rufini S.; Sette M.; Morero R.; Grottesi A.; Paci M.; Petruzzelli R. Zn2+ Ions Selectively Induce Antimicrobial Salivary Peptide Histatin-5 To Fuse Negatively Charged Vesicles. Identification and Characterization of a Zinc-Binding Motif Present in the Functional Domain. Biochemistry 1999, 38, 9626–9633. 10.1021/bi990212c. 10423240

[ref35] Singwi K.; Sjölander A. Diffusive Motions in Water and Cold Neutron Scattering. Phys. Rev. 1960, 119, 863–871. 10.1103/PhysRev.119.863.

[ref36] Cragnell C.; Durand D.; Cabane B.; Skepö M. Coarse-grained modeling of the intrinsically disordered protein Histatin 5 in solution: Monte Carlo simulations in combination with SAXS. Proteins: Struct., Funct., Bioinf. 2016, 84, 777–791. 10.1002/prot.25025. 26914439

[ref37] Fagerberg E.; Lenton S.; Skepö M. Evaluating Models of Varying Complexity of Crowded Intrinsically Disordered Protein Solutions Against SAXS. J. Chem. Theory Comput. 2019, 15, 6968–6983. 10.1021/acs.jctc.9b00723. 31714774

[ref38] Raj P. A.; Marcus E.; Sukumaran D. K. Structure of human salivary histatin 5 in aqueous and nonaqueous solutions. Biopolymers 1998, 45, 51–67. 10.1002/(SICI)1097-0282(199801)45:1<51::AID-BIP5>3.0.CO;2-Y. 9433185

[ref39] Brewer D.; Hunter H.; Lajoie G. NMR studies of the antimicrobial salivary peptides histatin 3 and histatin 5 in aqueous and nonaqueous solutions. Biochem. Cell Biol. 1998, 76, 247–256. 10.1139/o98-066. 9923693

[ref40] Raj P. A.; Edgerton M.; Levine M. J. Salivary histatin 5: dependence of sequence, chain length, and helical conformation for candidacidal activity. J. Biol. Chem. 1990, 265, 3898–3905. 10.1016/S0021-9258(19)39678-4. 2406266

[ref41] Jephthah S.; Staby L.; Kragelund B. B.; Skepö M. Temperature Dependence of Intrinsically Disordered Proteins in Simulations: What are We Missing?. J. Chem. Theory Comput. 2019, 15, 2672–2683. 10.1021/acs.jctc.8b01281. 30865820

[ref42] Henriques J.; Cragnell C.; Skepö M. Molecular Dynamics Simulations of Intrinsically Disordered Proteins: Force Field Evaluation and Comparison with Experiment. J. Chem. Theory Comput. 2015, 11, 3420–3431. 10.1021/ct501178z. 26575776

[ref43] Henriques J.; Skepö M. Molecular Dynamics Simulations of Intrinsically Disordered Proteins: On the Accuracy of the TIP4P-D Water Model and the Representativeness of Protein Disorder Models. J. Chem. Theory Comput. 2016, 12, 3407–3415. 10.1021/acs.jctc.6b00429. 27243806

[ref44] Liu H.; Song D.; Zhang Y.; Yang S.; Luo R.; Chen H.-F. Extensive tests and evaluation of the CHARMM36IDPSFF force field for intrinsically disordered proteins and folded proteins. Phys. Chem. Chem. Phys. 2019, 21, 21918–21931. 10.1039/C9CP03434J. 31552948 PMC7198049

[ref45] de Souza J. V.; Zariquiey F. S.; Bronowska A. K. Development of Charge-Augmented Three-Point Water Model (CAIPi3P) for Accurate Simulations of Intrinsically Disordered Proteins. Int. J. Mol. Sci. 2020, 21, 6166 10.3390/ijms21176166. PMC7504337 32859072

[ref46] Sullivan S. S.; Weinzierl R. O. Optimization of Molecular Dynamics Simulations of c-MYC1–88—An Intrinsically Disordered System. Life 2020, 10, 109 10.3390/life10070109. PMC7400636 32664335

[ref47] Skepö M.; Cragnell C.; Nylander T.; Ollivier J.; Seydel T. To understand the antimicrobial activity of the salivary protein Histatin 5; Experiment Data, Institut Laue-Langevin (ILL): 2017; 10.5291/ILL-DATA.8-04-790.

[ref48] Skepö M.; Cragnell C.; Fagerberg E.; Koza M. M.; Nylander T.; Seydel T. To understand the antimicrobial activity of the salivary protein Histatin 5; Experiment Data, Institut Laue-Langevin (ILL): 2018; 10.5291/ILL-DATA.8-04-813.

[ref49] Skepö M.; Appel M.; Fagerberg E.; Lenton S.; Nylander T.; Ollivier J.; Seydel T. To understand the antimicrobial activity of the salivary protein Histatin 5; Experiment Data, Institut Laue-Langevin (ILL): 2018; 10.5291/ILL-DATA.8-04-868.

[ref50] Frick B.; Mamontov E.; van Eijck L.; Seydel T. Recent backscattering instrument developments at the ILL and SNS. Z. Chem. (Stuttgart, Ger.) 2010, 224, 33–60. 10.1524/zpch.2010.6091.

[ref51] Hennig M.; Frick B.; Seydel T. Optimum velocity of a phase-space transformer for cold-neutron backscattering spectroscopy. J. Appl. Crystallogr. 2011, 44, 467–472. 10.1107/S0021889811013227.

[ref52] Arnold O.; Bilheux J.; Borreguero J.; Buts A.; Campbell S.; Chapon L.; Doucet M.; Draper N.; Ferraz Leal R.; Gigg M.; et al. Mantid—Data analysis and visualization package for neutron scattering and μ SR experiments. Nuclear Instruments and Methods in Physics Research Section A: Accelerators, Spectrometers, Detectors and Associated Equipment 2014, 764, 156–166. 10.1016/j.nima.2014.07.029.

[ref53] Cohn E.; Edsall J. Proteins, Amino Acids and Peptides as Ions and Dipolar Ions; Reinhold Publishing Corporation: 1943; Chapter 4, p 157.

[ref54] Cho C.; Urquidi J.; Singh S.; Robinson G. W. Thermal offset viscosities of liquid H2O, D2O, and T2O. J. Phys. Chem. B 1999, 103, 1991–1994. 10.1021/jp9842953.

[ref55] Paalman H. H.; Pings C. J. Numerical Evaluation of X-Ray Absorption Factors for Cylindrical Samples and Annular Sample Cells. J. Appl. Phys. (Melville, NY, U. S.) 1962, 33, 2635–2639. 10.1063/1.1729034.

[ref56] Augé S.; Schmit P.-O.; Crutchfield C. A.; Islam M. T.; Harris D. J.; Durand E.; Clemancey M.; Quoineaud A.-A.; Lancelin J.-M.; Prigent Y.; et al. NMR Measure of Translational Diffusion and Fractal Dimension. Application to Molecular Mass Measurement. J. Phys. Chem. B 2009, 113, 1914–1918. 10.1021/jp8094424. 19173563

[ref57] Ahmed M. C.; Skaanning L. K.; Jussupow A.; Newcombe E. A.; Kragelund B. B.; Camilloni C.; Langkilde A. E.; Lindorff-Larsen K. Refinement of α-Synuclein Ensembles Against SAXS Data: Comparison of Force Fields and Methods. Front. Mol. Biosci. 2021, 8, 216 10.3389/fmolb.2021.654333. PMC8100456 33968988

[ref58] Johansen D.; Trewhella J.; Goldenberg D. P. Fractal dimension of an intrinsically disordered protein: Small-angle X-ray scattering and computational study of the bacteriophage λ N protein. Protein Sci. 2011, 20, 1955–1970. 10.1002/pro.739. 21936008 PMC3302640

[ref59] Jephthah S.; Pesce F.; Lindorff-Larsen K.; Skepö M. Force Field Effects in Simulations of Flexible Peptides with Varying Polyproline II Propensity. J. Chem. Theory Comput. 2021, 17, 6634 10.1021/acs.jctc.1c00408. 34524800 PMC8515809

[ref60] Berendsen H.; van der Spoel D.; van Drunen R. GROMACS: A message-passing parallel molecular dynamics implementation. Comput. Phys. Commun. 1995, 91, 43–56. 10.1016/0010-4655(95)00042-E.

[ref61] Lindahl E.; Hess B.; van der Spoel D. GROMACS 3.0: a package for molecular simulation and trajectory analysis. J. Mol. Model. 2001, 7, 306–317. 10.1007/s008940100045.

[ref62] van Der Spoel D.; Lindahl E.; Hess B.; Groenhof G.; Mark A. E.; Berendsen H. J. C. GROMACS: Fast, flexible, and free. J. Comput. Chem. 2005, 26, 1701–1718. 10.1002/jcc.20291. 16211538

[ref63] Hess B.; Kutzner C.; van der Spoel D.; Lindahl E. GROMACS 4: Algorithms for Highly Efficient, Load-Balanced, and Scalable Molecular Simulation. J. Chem. Theory Comput. 2008, 4, 435–447. 10.1021/ct700301q. 26620784

[ref64] Abraham M. J.; Murtola T.; Schulz R.; Páll S.; Smith J. C.; Hess B.; Lindahl E. GROMACS: High performance molecular simulations through multi-level parallelism from laptops to supercomputers. SoftwareX 2015, 1–2, 19–25. 10.1016/j.softx.2015.06.001.

[ref65] Robustelli P.; Piana S.; Shaw D. E. Developing a molecular dynamics force field for both folded and disordered protein states. Proc. Natl. Acad. Sci. U. S. A. 2018, 115, E4758–E4766. 10.1073/pnas.1800690115. 29735687 PMC6003505

[ref66] Shrestha U.; Smith J.; Petridis L. Full structural ensembles of intrinsically disordered proteins from unbiased molecular dynamics simulations. Commun. Biol. 2021, 4, 243 10.1038/s42003-021-01759-1. 33623120 PMC7902620

[ref67] Bremer A.; Wolff M.; Thalhammer A.; Hincha D. K. Folding of intrinsically disordered plant LEA proteins is driven by glycerol-induced crowding and the presence of membranes. FEBS J. 2017, 284, 919–936. 10.1111/febs.14023. 28109185

[ref68] Hess B.; Bekker H.; Berendsen H. J. C.; Fraaije J. G. E. M. LINCS: A linear constraint solver for molecular simulations. J. Comput. Chem. 1997, 18, 1463–1472. 10.1002/(SICI)1096-987X(199709)18:12<1463::AID-JCC4>3.0.CO;2-H.

[ref69] Darden T.; York D.; Pedersen L. Particle mesh Ewald: An N log(N) method for Ewald sums in large systems. J. Chem. Phys. 1993, 98, 10089–10092. 10.1063/1.464397.

[ref70] Bussi G.; Donadio D.; Parrinello M. Canonical sampling through velocity rescaling. J. Chem. Phys. 2007, 126, 014101 10.1063/1.2408420. 17212484

[ref71] Goret G.; Aoun B.; Pellegrini E. MDANSE: An Interactive Analysis Environment for Molecular Dynamics Simulations. J. Chem. Inf. Model. 2017, 57, 1–5. 10.1021/acs.jcim.6b00571. 28026944

[ref72] von Bülow S.; Siggel M.; Linke M.; Hummer G. Dynamic cluster formation determines viscosity and diffusion in dense protein solutions. Proc. Natl. Acad. Sci. U. S. A. 2019, 116, 9843–9852. 10.1073/pnas.1817564116. 31036655 PMC6525548

[ref73] Nawrocki G.; Wang P.; Yu I.; Sugita Y.; Feig M. Slow-Down in Diffusion in Crowded Protein Solutions Correlates with Transient Cluster Formation. J. Phys. Chem. B 2017, 121, 11072–11084. 10.1021/acs.jpcb.7b08785. 29151345 PMC5951686

[ref74] Yeh I.-C.; Hummer G. System-Size Dependence of Diffusion Coefficients and Viscosities from Molecular Dynamics Simulations with Periodic Boundary Conditions. J. Phys. Chem. B 2004, 108, 15873–15879. 10.1021/jp0477147.

[ref75] Hasimoto H. On the periodic fundamental solutions of the Stokes equations and their application to viscous flow past a cubic array of spheres. J. Fluid Mech. 1959, 5, 317–328. 10.1017/S0022112059000222.

[ref76] Ong E. E.; Liow J.-L. The temperature-dependent structure, hydrogen bonding and other related dynamic properties of the standard TIP3P and CHARMM-modified TIP3P water models. Fluid Phase Equilib. 2019, 481, 55–65. 10.1016/j.fluid.2018.10.016.

[ref77] Hess B. Determining the shear viscosity of model liquids from molecular dynamics simulations. J. Chem. Phys. 2002, 116, 209–217. 10.1063/1.1421362.

[ref78] Ortega A.; Amoros D.; García de la Torre J. Prediction of Hydrodynamic and Other Solution Properties of Rigid Proteins from Atomic- and Residue-Level Models. Biophys. J. 2011, 101, 892–898. 10.1016/j.bpj.2011.06.046. 21843480 PMC3175065

[ref79] Nygaard M.; Kragelund B. B.; Papaleo E.; Lindorff-Larsen K. An Efficient Method for Estimating the Hydrodynamic Radius of Disordered Protein Conformations. Biophys. J. 2017, 113, 550–557. 10.1016/j.bpj.2017.06.042. 28793210 PMC5550300

[ref80] Goldsack D. E.; Franchetto R. The viscosity of concentrated electrolyte solutions. I. Concentration dependence at fixed temperature. Can. J. Chem. 1977, 55, 1062–1072. 10.1139/v77-148.

[ref81] Goldsack D. E.; Franchetto R. The viscosity of concentrated electrolyte solutions. 11. Temperature dependence. Can. J. Chem. 1978, 56, 1442–1450. 10.1139/v78-236.

[ref82] Grimaldo M.; Roosen-Runge F.; Hennig M.; Zanini F.; Zhang F.; Zamponi M.; Jalarvo N.; Schreiber F.; Seydel T. Salt-Induced Universal Slowing Down of the Short-Time Self-Diffusion of a Globular Protein in Aqueous Solution. J. Phys. Chem. Lett. 2015, 6, 2577–2582. 10.1021/acs.jpclett.5b01073. 26266736

[ref83] Berg H. C. Random Walks in Biology; Princeton University Press: Princeton, NJ, 1993.

[ref84] Tokuyama M.; Oppenheim I. Dynamics of hard-sphere suspensions. Phys. Rev. E 1994, 50, R16–R19. 10.1103/PhysRevE.50.R16. 9962019

[ref85] Medina-Noyola M. Long-Time Self-Diffusion in Concentrated Colloidal Dispersions. Phys. Rev. Lett. 1988, 60, 2705–2708. 10.1103/PhysRevLett.60.2705. 10038430

[ref86] Roosen-Runge F.; Schurtenberger P.; Stradner A. Self-diffusion of nonspherical particles fundamentally conflicts with effective sphere models. J. Phys.: Condens. Matter 2021, 33, 154002 10.1088/1361-648X/abdff9. 33498038

[ref87] Perticaroli S.; Ehlers G.; Stanley C. B.; Mamontov E.; O’Neill H.; Zhang Q.; Cheng X.; Myles D. A.; Katsaras J.; Nickels J. D. Description of hydration water in protein (green fluorescent protein) solution. J. Am. Chem. Soc. 2017, 139, 1098–1105. 10.1021/jacs.6b08845. 27783480

[ref88] Henriques J.; Arleth L.; Lindorff-Larsen K.; Skepö M. On the Calculation of SAXS Profiles of Folded and Intrinsically Disordered Proteins from Computer Simulations. J. Mol. Biol. 2018, 430, 2521–2539. 10.1016/j.jmb.2018.03.002. 29548755

[ref89] Wang Y.; Benton L. A.; Singh V.; Pielak G. J. Disordered Protein Diffusion under Crowded Conditions. J. Phys. Chem. Lett. 2012, 3, 2703–2706. 10.1021/jz3010915. 23185649 PMC3505085

