# Supplementary material for: Self-Diffusive Properties of the Intrinsically Disordered Protein Histatin 5 and the Impact of Crowding Thereon: A Combined Neutron Spectroscopy and Molecular Dynamics Simulation Study
Source: J Phys Chem B. 2022 Jan 19;126(4):789–801. doi: 10.1021/acs.jpcb.1c08976 (PMC8819652; doi:10.1021/acs.jpcb.1c08976)
Supplement: Supplementary file 1 — jp1c08976_si_001.pdf [file jp1c08976_si_001.pdf]

Supporting Information of "The Self-Diffusive Properties of the Intrinsically Disordered Protein Histatin 5 and the Impact of Crowding Thereon: A Combined Neutron Spectroscopy and Molecular Dynamics Simulation Study"

Eric Fagerberg,[†] Samuel Lenton,[‡] Tommy Nylander,[‡] Tilo Seydel,[¶] and Marie Skepö[*,†,§]

†*Theoretical Chemistry, Lund University, POB 124, SE-221 00 Lund, Sweden*

‡*Physical Chemistry, Lund University, POB 124, SE-221 00 Lund, Sweden*

¶*Institut Max von Laue - Paul Langevin, 71 avenue des Martyrs, CS 20156, F-38042 Grenoble, France.*

§*LINXS - Lund Institute of Advanced Neutron and X-ray Science, Scheelevägen 19, SE-223 70 Lund, Sweden*

E-mail: marie.skepo@teokem.lu.se

1 Samples measured

Table S1: All the samples measured using QENS, specifying protein concentration, temperature, amount of salt (NaCl) and whether sample was dialyzed before measurement.

Protein,Conc	Temp	Salt	Dialyzed?	Numbering
Hst5, 50 mg/ml	280 K	150 mM	Yes	1
Hst5, 50 mg/ml	298 K	150 mM	Yes	2
Hst5 100 mg/ml	280 K	150 mM	Yes	3
Hst5, 100 mg/ml	298 K	150 mM	Yes	4
Hst5, 100 mg/ml	310 K	150 mM	Yes	5
Hst5, 150 mg/ml	280 K	150 mM	Yes	6
Hst5, 150 mg/ml	290 K	150 mM	Yes	7
Hst5, 150 mg/ml	298 K	150 mM	Yes	8
Hst5, 150 mg/ml	310 K	150 mM	Yes	9
Hst5, 185 mg/ml	280 K	10 mM	Yes	10
Hst5, 185 mg/ml	298 K	10 mM	Yes	11
Hst5, 185 mg/ml	310 K	10 mM	Yes	12
Hst5, 200 mg/ml	280 K	150 mM	Yes	13
Hst5, 200 mg/ml	298 K	150 mM	Yes	14
Hst5, 200 mg/ml	310 K	150 mM	Yes	15
Hst5, 200 mg/ml	280 K	10 mM	No	16
Hst5, 200 mg/ml	298 K	10 mM	No	17
Hst5, 200 mg/ml	310 K	10 mM	No	18
Hst5, 200 mg/ml	280 K	150 mM	No	19
Hst5, 200 mg/ml	298 K	150 mM	No	20
Hst5, 200 mg/ml	310 K	150 mM	No	21
Hst5, 350 mg/ml	280 K	10 mM	No	22
Hst5, 350 mg/ml	298 K	10 mM	No	23
Hst5, 350 mg/ml	310 K	10 mM	No	24
Hst5 500 mg/ml	280 K	10 mM	No	25
Hst5, 500 mg/ml	298 K	10 mM	No	26
Hst5, 500 mg/ml	310 K	10 mM	No	27

2 QENS-spectra examples

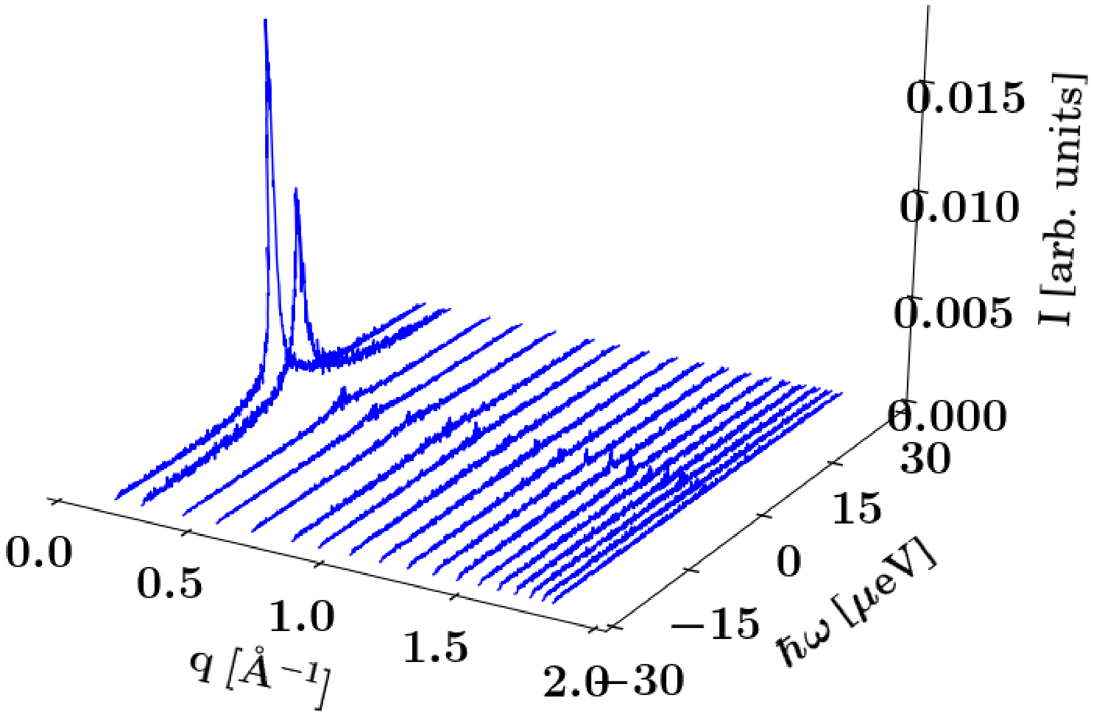

Figure S1: All the QENS-spectra at different q-values for Hst5 at 50 mg/ml protein concentration, 150 mM NaCl concentration and 298 K temperature.

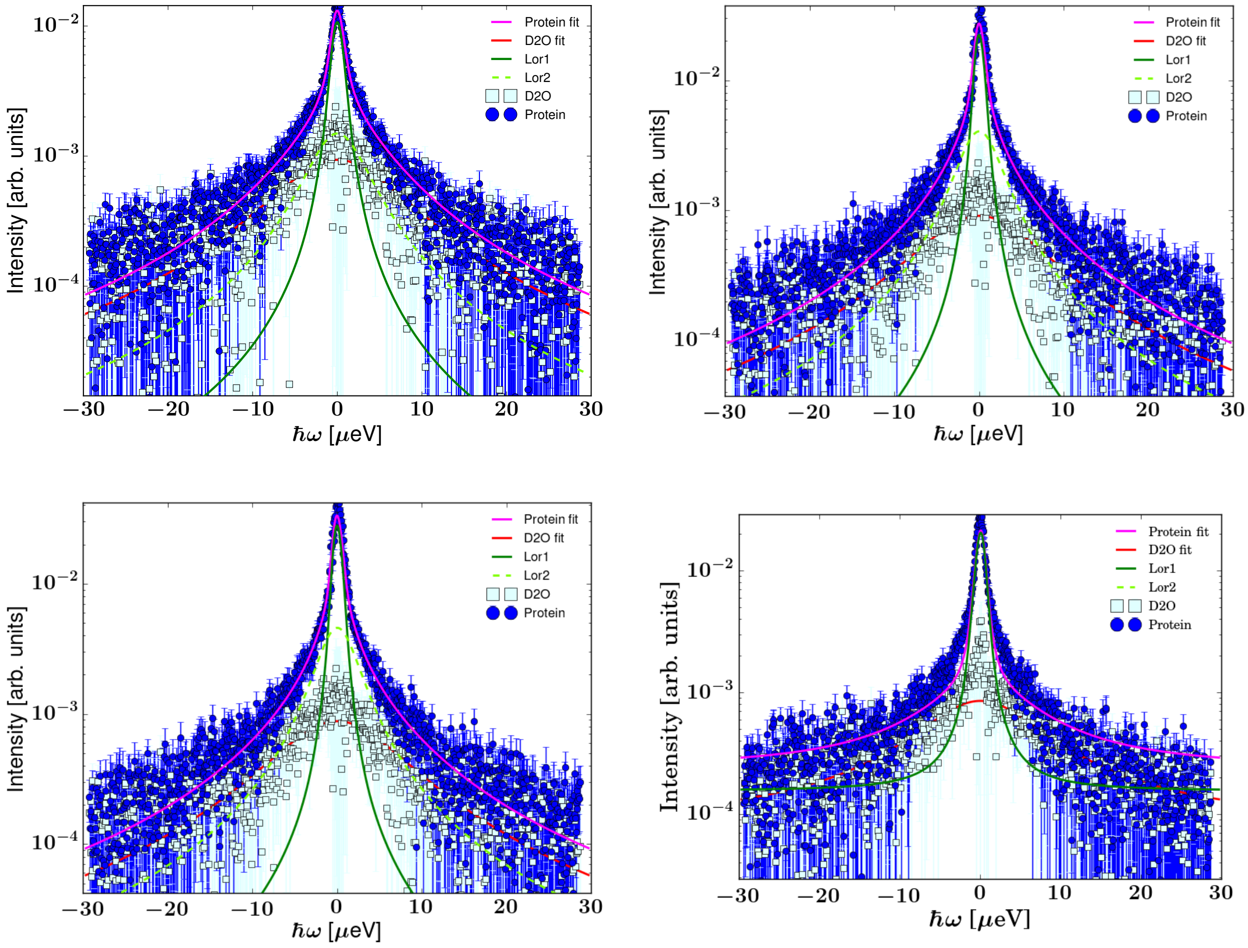

Figure S2: QENS-spectra at different protein concentrations at 280 K temperature, 150 mM NaCl concentration, q-value of 0.29 Å^{-1}, including Paalman-Pings corrections. From top left to bottom right right: 50 mg/ml, 100 mg/ml, 150 mg/ml, and 200 mg/ml protein concentration, respectively. Fitting is done with the "jump-diffusion" model, with one lorentzian for the solvent data and two lorentzians for the protein data ('Lor1' and 'Lor2').

2.1 EISF at different protein concentrations

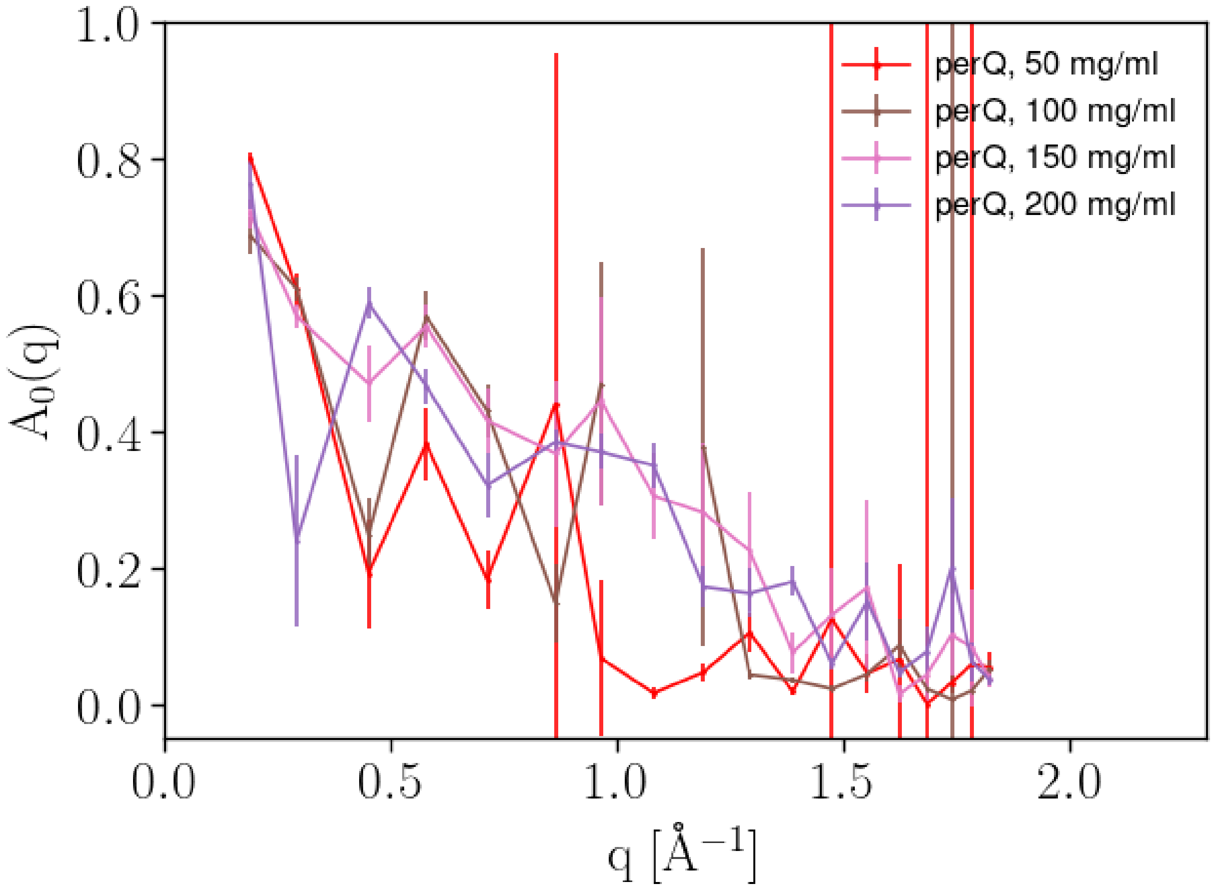

Figure S3: The elastic incoherent structure factor (EISF/A_0) for different protein concentrations at a temperature of 298 K and salt concentration of 150 mM NaCl. EISF obtained through fitting of the "per-q" model. Paalman-Pings corrections applied.

3 Fitting considerations

First it was evaluated as to whether Paalman-Pings corrections and/or a restricted q-range could improve fitting. In this case, a jump-diffusion model was used for internal diffusivity, with a dependence on momentum transfer, but it is assumed that results would be transferable to other models. Looking at goodness-of-fit (Figure S1), there is evidence that Paalman-Pings corrections have a positive impact on goodness-of-fit, with some exception of the low-concentration samples. However, this may be due to an increase in error, which

would cause the goodness-of-fit to decrease, which, depending on the difference in fitted and experimental values, may cause a bad fit to be categorized as good. Therefore, L1/L2 loss functions are also considered, see Figures S2 and S3.

It is seen that for samples with lower protein concentration and that have been dialyzed, L1 and L2 loss functions indicate Paalman-Pings corrections to indeed improve fitting. For these samples, there is a very small, negative impact of restricting the q-range. However, for higher protein concentration and non-dialyzed samples, L1/L2 metrics indicate fits to be worsened by Paalman-Pings corrections. The non-dialyzed samples will be considered separately, therefore the continued discussion concern fittings with Paalman-Pings corrections and a full q-range. For illustration of the impact of the fits, computed apparent diffusion using Paalman-Pings corrected data and empty-can subtracted data is 0.674 Å^2/ns and 0.518 Å^2/ns, respectively, for the sample with the largest difference in fitting metrics (#25). Two models were compared, one with the Singwi-Sjölander jump-diffusion model for internal diffusivity which fits imposes a momentum transfer dependence (across all q simultaneously) denoted "Jump-diffusion" and a Fickian model $\gamma = Dq^2$ fitting a Lorentzian for each q individually, denoted "per-q". As can be seen from Figures S4 and S5, resulting fits are very much comparable, with a minor advantage for per-q fitting. However, this overview excludes the fact that for some individual q-values, the per-q fitting failed. Given the high similarity between the models in terms of adequate fitting, the failure of the per-q model in a few cases (in total five cases across all samples) determines the choice of mainly analysing results with the jump-diffusion model, which give more stable fits in terms of center-of-mass diffusion. Extracting a diffusion constant from the per-q model is as well not always tractable; using the $\gamma = Dq^2$ relation will sometimes yield poor correlation coefficient.

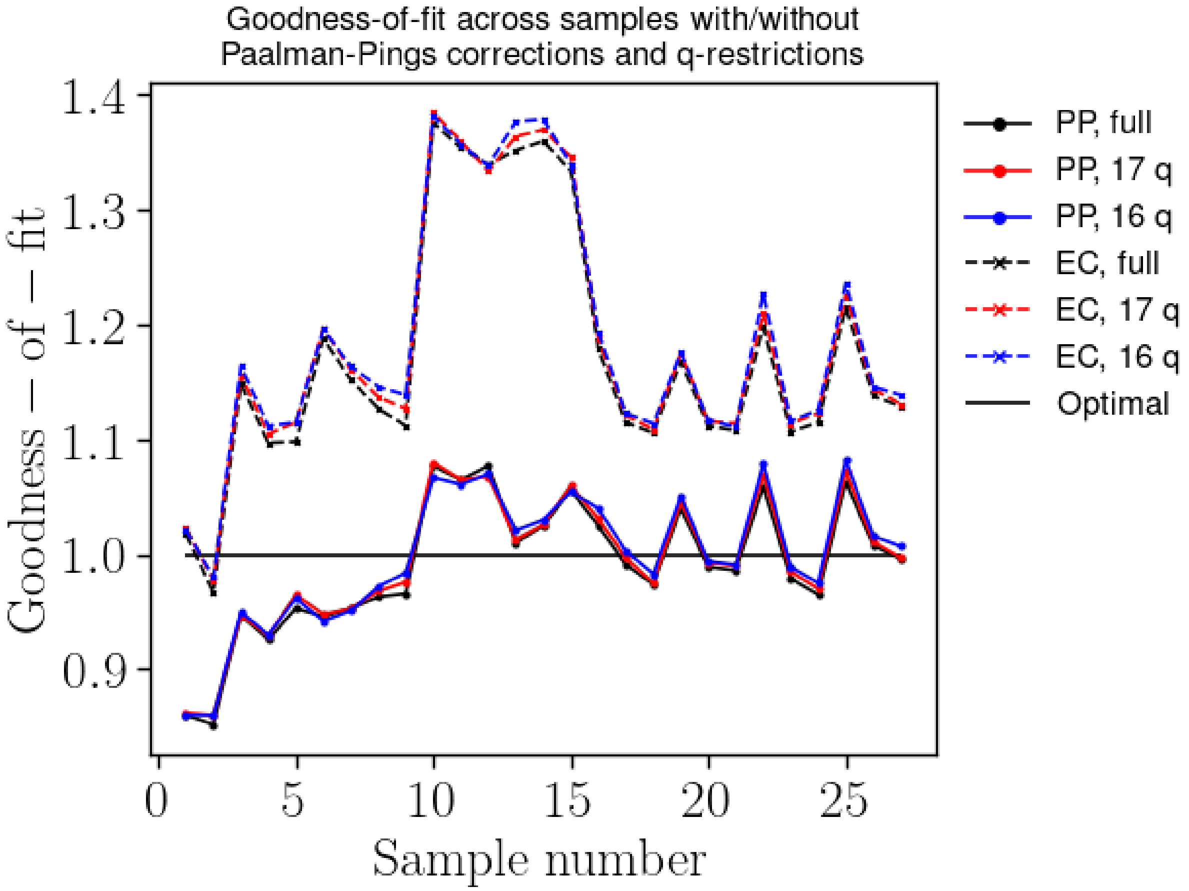

Figure S4: Goodness-of-fit for all samples measured.

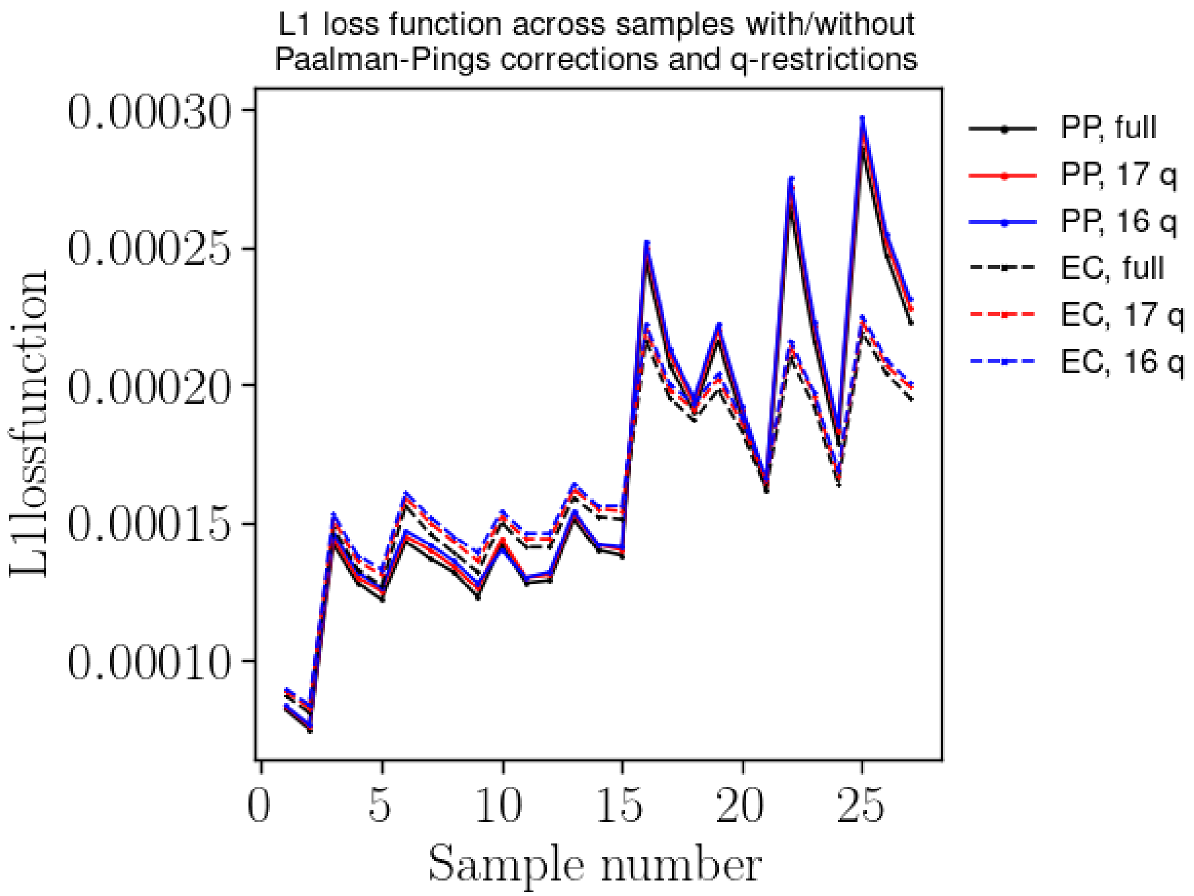

Figure S5: L1-loss function for all Hst5 samples.

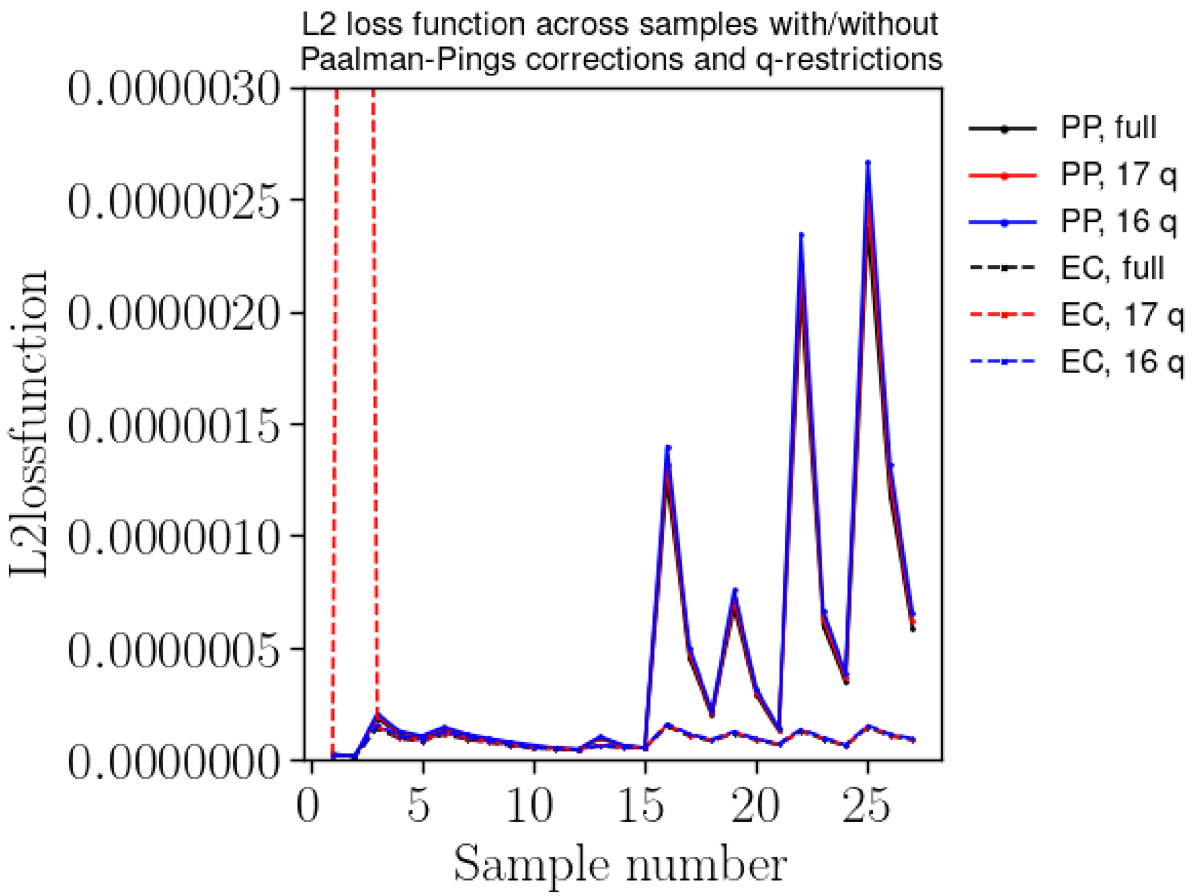

Figure S6: L2-loss function for all Hst5 samples.

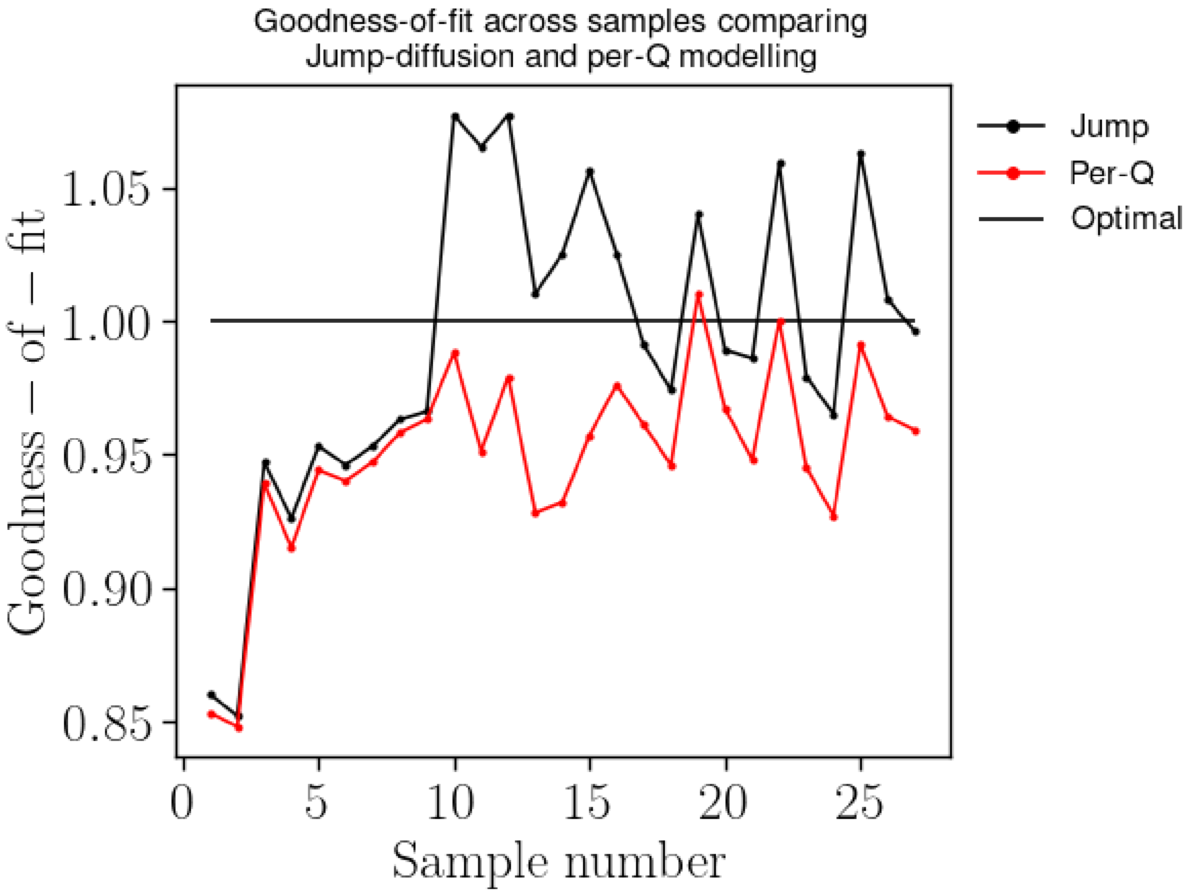

Figure S7: Goodness-of-fit comparing jump-diffusion and per-q models.

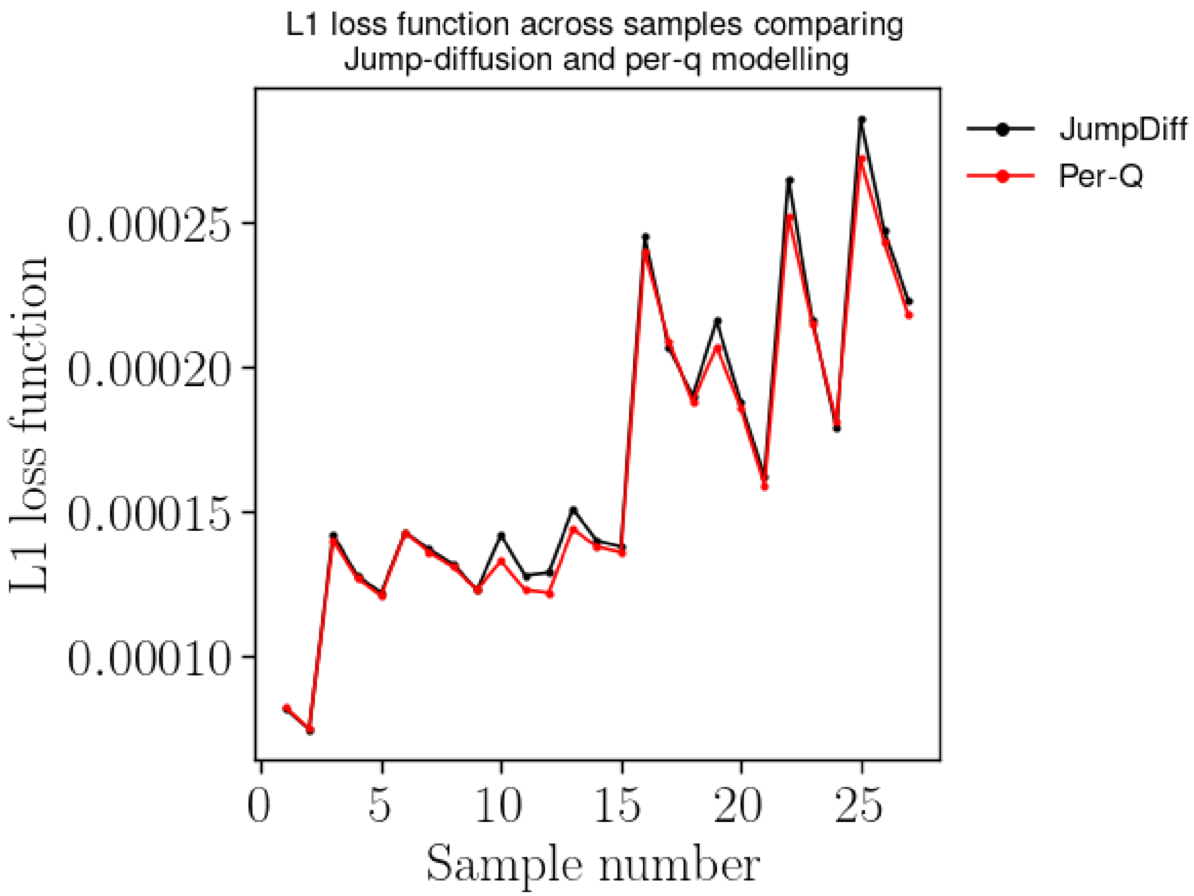

Figure S8: L1-loss function comparing jump-diffusion and per-q models.

4 Samples with varying salt content

The proteins used in this study are synthesised, and the manufacturer of the samples has communicated that there is non-protein content in the samples, mainly TFA (CF_3COONa) and other monovalent salts (e.g. NaCl, KCl), which on average is 35 % of the content. Two extreme cases are identified: Either all salt is TFA, or all salt is NaCl, considering the least and largest amount of salt molecules available (KCl weighs in between and would not be an extreme case in terms of number of molecules in the solution). The molar concentration of salt is computed for these two extremes: In case of all salt being NaCl, a (monovalent) salt concentration of 5.15, 3.42, 1.9, 2.05 M for the 500, 350, and 200 mg/ml in "10 mM salt" and 200 mg/ml in "150 mM" salt, respectively. In case of all salt being TFA, the

corresponding numbers are 2.21, 1.47, 0.84, and 0.99 M, respectively. These observations indicate the importance of careful sample preparation. In this study, an assumption of all salt being NaCl is made, as parameters for viscosity change computation are not available for TFA (as far as we are aware).

Equation according to Goldsack and Franchetto[1]:

$$\eta = \frac{\eta_1 e^{XE}}{1 + XV} \tag{1}$$

where X is the mole fraction of cations, E is a free energy and V is a volume (see reference for details). η_1 is taken as the viscosity of D2O at the corresponding temperature, as computed from the relation by Cho *et al.*[2]

4.1 Assuming all salt is NaCl

Parameters E, V for NaCl for different temperatures are found in Goldsack and Franchetto.[3]

Table S2: Computation of viscosity due to excessive salt in unpurified samples.

Sample	Temp [K]	Computed Viscosity [Pa S]
500 mg/ml	280	0.00333
350 mg/ml	280	0.00263
200 mg/ml "10mM salt"	280	0.00218
200 mg/ml "150mM salt"	280	0.00222
500 mg/ml	298	0.00205
350 mg/ml	298	0.00161
200 mg/ml "10mM salt"	298	0.00133
200 mg/ml "150mM salt"	298	0.00135
500 mg/ml	310	0.00155
350 mg/ml	310	0.00123
200 mg/ml "10mM salt"	310	0.00101
200 mg/ml "150mM salt"	310	0.00103

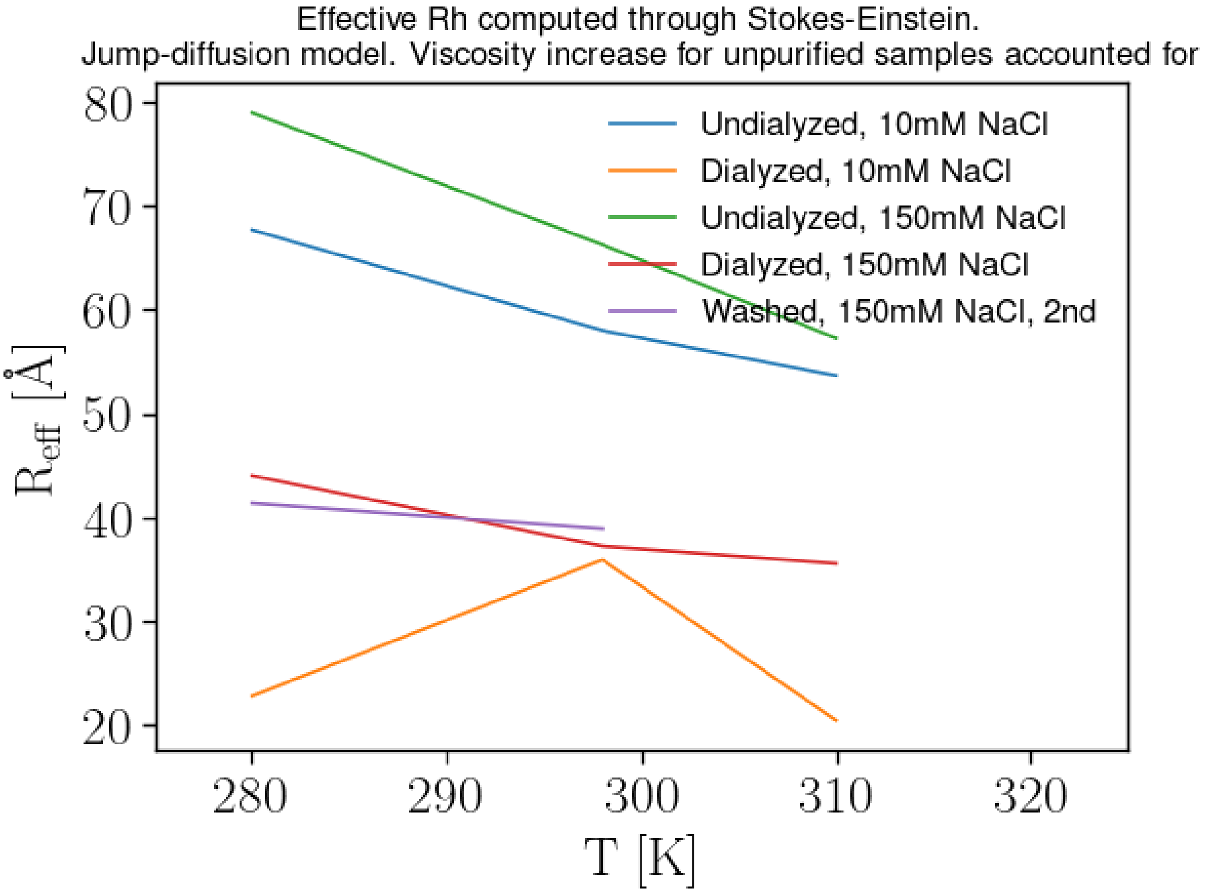

Figure S9: Comparison of effective radius of hydration computed from the effective diffusion, comparing different sample preparation methods, which gives different amounts of salt content in the sample.

5 Figures for parameters using jump-diffusion model

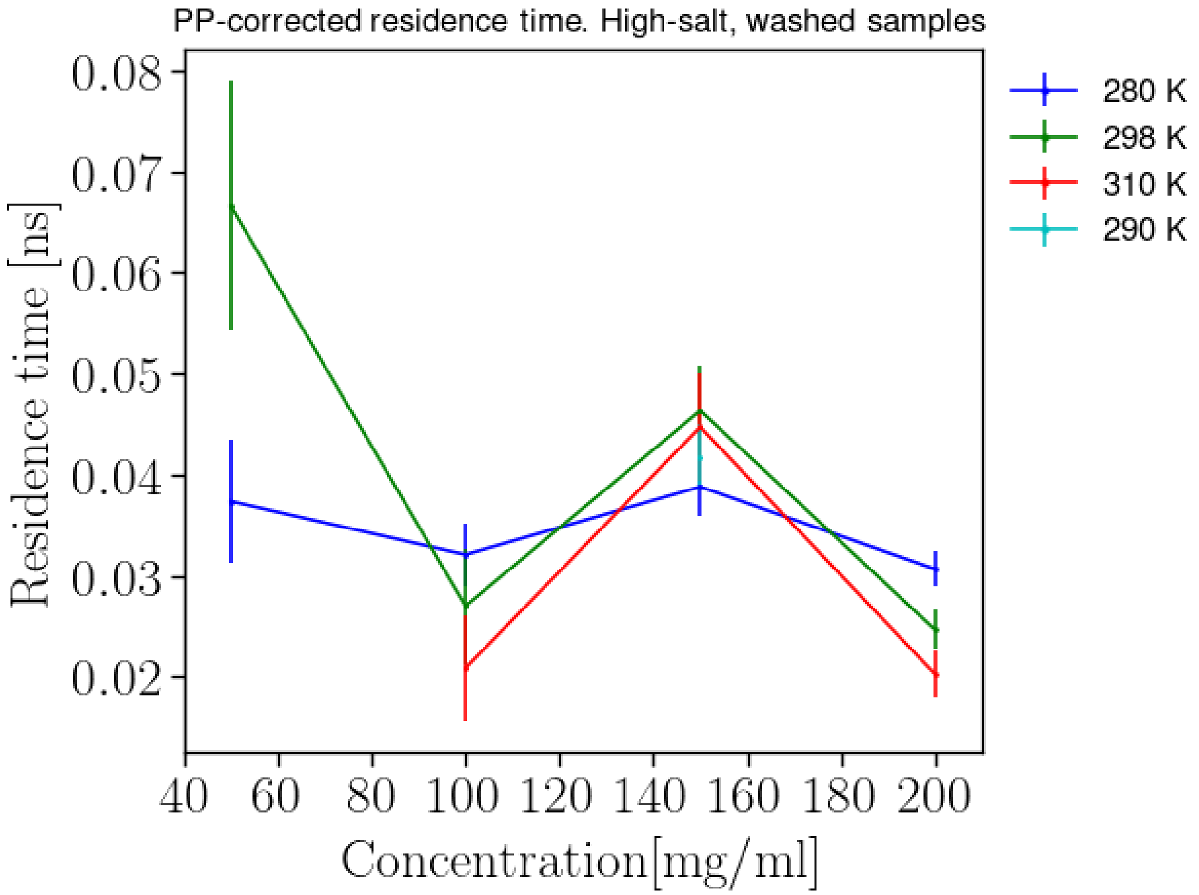

Figure S10: Residence time (t1) for the jump-diffusion model for different samples. Data includes Paalman-Pings corrections.

Table S3: Parameters jump-diffusion model. D is the apparent (Fickian) center-of-mass diffusion, D1 is the internal (jump) diffusion, t1 is the residence time.

Sample	D	D1	t1
1	8.45 +/- 0.427	60.8 +/- 4.67	0.0374 +/- 0.00603
2	16.8 +/- 0.659	388 +/- 83.1	0.00666 +/- 0.0124
3	5.01 +/- 0.155	45.2 +/- 2.12	0.0321 +/- 0.00312
4	10.4 +/- 0.333	107 +/- 6.31	0.0270 +/- 0.00568
5	13.8 +/- 0.423	180 +/- 10.5	0.0209 +/- 0.0052
6	4.19 +/- 0.111	42.4 +/- 1.91	0.0388 +/- 0.0028
7	6.01 +/- 0.178	73.3 +/- 3.40	0.0417 +/- 0.00324
8	8.00 +/- 0.263	102 +/- 5.76	0.0464 +/- 0.0045
9	11.9 +/- 0.373	183 +/- 11.9	0.0447 +/- 0.0053
10	4.82 +/- 0.0752	840561399 +/- 0.0	0.0430 +/- 0.00207
11	5.50 +/- 0.175	49.9 +/- 2.38	0.0188 +/- 0.00232
12	13.2 +/- 0.259	2860 +/- 1320	0.0119 +/- 0.00549
13	2.50 +/- 0.0478	28.1 +/- 0.970	0.0307 +/- 0.00169
14	5.31 +/- 0.120	66.3 +/- 2.63	0.0247 +/- 0.00196
15	7.60 +/- 0.189	106 +/- 5.09	0.0203 +/- 0.00231
16	1.39 +/- 0.200	22.1 +/- 0.660	0.0271 +/- 0.00168
17	2.83 +/- 0.0482	38.7 +/- 1.26	0.0217 +/- 0.00178
18	4.19 +/- 0.0835	58.5 +/- 2.03	0.0199 +/- 0.00196
19	1.17 +/- 0.0214	18.3 +/- 0.644	0.0355 +/- 0.00213
20	2.44 +/- 0.0509	29.2 +/- 1.11	0.0211 +/- 0.00204
21	3.85 +/- 0.0882	47.6 +/- 1.81	0.0206 +/- 0.00203
22	0.564 +/- 0.00801	13.8 +/- 0.426	0.0462 +/- 0.00219
23	1.78 +/- 0.0248	31.7 +/- 1.06	0.0259 +/- 0.00166
24	2.59 +/- 0.0385	45.3 +/- 1.45	0.0274 +/- 0.00148
25	0.674 +/- 0.00874	17.8 +/- 0.536	0.0424 +/- 0.00179
26	1.37 +/- 0.0183	31.7 +/- 0.976	0.0349 +/- 0.00144
27	2.11 +/- 0.0314	43.6 +/- 1.45	0.0322 +/- 0.00152

6 MDANSE calculation

The software MDANSE[4] was used to calculate the EISF from the trajectories. According to the internal documentation of the program, this computation is achieved by a using a grid of equidistantly spaced points along the q-axis, according to Eq. 2:

$$EISF(q_m) = \sum_{I=1}^{N_{species}} n_I \omega_I EISF_I(q_m), m = 0...N_q - 1 \quad (2)$$

where n_I is the number of atoms of specie I, ω_I is the weight for specie I, N_q is a

user-defined number of shells and $q_m = q_{min} + m * \Delta q$. $EISF_I(q_m)$ is defined in Eq. 3

$$EISF_I(q_m) = \frac{1}{n_I} \sum_{\alpha}^{n_I} \overline{\langle |exp[i\mathbf{q}\mathbf{R}_\alpha]|^2 \rangle}^q \tag{3}$$

where the overbar-q denotes that it is an average over the q-values having the same modulus q_m and R_α is the position of particle α.

7 Comparison with molecular dynamics simulation

7.1 Single-chain simulation: A99SBN-ILDN

Intermediary numbers found in the calculation of the effective diffusion from the molecular dynamics simulation performed by Henriques *et al.*[5]. Note that convergence information on this simulation is found in the original article by Henriques *et al.*

Table S4: Diffusion data for each replicate in the simulation by Henriques *et al.* In this table, data is NOT corrected for finite-size effects or the water/deuterium difference. The number after the ± sign refers to the error of the linear fit to mean-square displacement.

Replicate	Trans. Diff. [Å^2/ns]
1	17.048 ± 0.035
2	17.604 ± 0.040
3	16.979 ± 0.032
4	17.292 ± 0.046
5	18.017 ± 0.031
Average	17.388
STD	0.38325

Translational diffusion after fix of finite-size effects: 27.673 Å^2 / ns.

After adjusting for the fact that simulation was done in water, while experiment was performed in deuterium, correcting for viscosity difference between the two: 22.394 Å^2 / ns, standard deviation 0.31014

Using Hydropro As input to HYDROPRO, the viscosity and density of D2O is given, rather than using the values from simulation which used H2O (when not using HYDROPRO,

this is adjusted for in post-processing). Snapshots from simulation were extracted every 100 ps. The following computed numbers (averaged across all replicates) were obtained:

Translational diffusion: 13.18, standard deviation 0.78 $Å^2$ / ns

Radius of gyration: 14.2 Å (Note that HYDROPRO assumes an atomic hydration layer of 1.1 Å in this computation).

No finite-size adjustment for diffusion is done in this case, as HYDROPRO is parametrized to directly compute properties from crystal structures.

7.2 Single-chain simulation: C36IDPS

Intermediary numbers found in the calculation of the effective diffusion from the molecular dynamics simulation performed by Jephthah *et al.*[6] Note that information about convergence of these trajectories is found in the original article.

Table S5: Diffusion data for each replicate in the simulation by Jephthah *et al.* In this table, data is NOT corrected for finite-size effects or the water/deuterium difference. The number after the ± sign refers to the error of the linear fit to mean-square displacement.

Replicate	Trans. Diff. [$Å^2$/ns]
1	45.633 ± 0.059
2	42.262 ± 0.096
3	43.873 ± 0.094
4	43.291 ± 0.091
5	43.868 ± 0.097
Average	43.785
STD	1.0948

Translational diffusion after fix of finite-size effects: 73.857 $Å^2$ / ns.

After adjusting for the fact that simulation was done in water, while experiment was performed in deuterium, correcting for viscosity difference between the two: 59.767 $Å^2$ / ns, standard deviation 0.88595

Using Hydropro As input to HYDROPRO, the viscosity and density of D2O is given, rather than using the values from simulation which used H2O (when not using HYDROPRO,

this is adjusted for in post-processing). Snapshots from simulation were extracted every 100 ps. The following computed numbers (averaged across all replicates) were obtained:

Translational diffusion: 13.94, standard deviation 0.87 Å^2 / ns

Radius of gyration: 12.6 Å (Note that HYDROPRO assumes an atomic hydration layer of 1.1 Å in this computation).

No finite-size adjustment for diffusion is done in this case, as HYDROPRO is parametrized to directly compute properties from crystal structures.

7.3 Single-chain simulation: C36m

Intermediary numbers found in the calculation of the effective diffusion from the molecular dynamics simulation performed by Jephthah *et al.*[6] Note that information about convergence of these trajectories is found in the original article.

Table S6: Diffusion data for each replicate in the simulation by Jephthah *et al.* using the CHARMM36m force field. In this table, data is NOT corrected for finite-size effects or the water/deuterium difference. The number after the ± sign refers to the error of the linear fit to mean-square displacement.

Replicate	Trans. Diff. [Å^2/ns]
1	41.576 ± 0.058
2	37.422 ± 0.102
3	38.020 ± 0.093
4	38.723 ± 0.077
5	37.420 ± 0.108
Average	38.632
STD	1.5481

Translational diffusion after fix of finite-size effects: 68.742 Å^2 / ns.

After adjusting for the fact that simulation was done in water, while experiment was performed in deuterium, correcting for viscosity difference between the two: 55.628 Å^2 / ns, standard deviation 1.2528

Using Hydropro As input to HYDROPRO, the viscosity and density of D2O is given, rather than using the values from simulation which used H2O (when not using HYDROPRO,

this is adjusted for in post-processing). Snapshots from simulation were extracted every 100 ps. The following computed numbers (averaged across all replicates) were obtained:

Translational diffusion: 13.18, standard deviation 0.93 Å^2 / ns

Radius of gyration: Å (Note that HYDROPRO assumes an atomic hydration layer of 1.1 Å in this computation).

No finite-size adjustment for diffusion is done in this case, as HYDROPRO is parametrized to directly compute properties from crystal structures.

7.4 Single-chain simulation: A99SB-disp

Convergence information

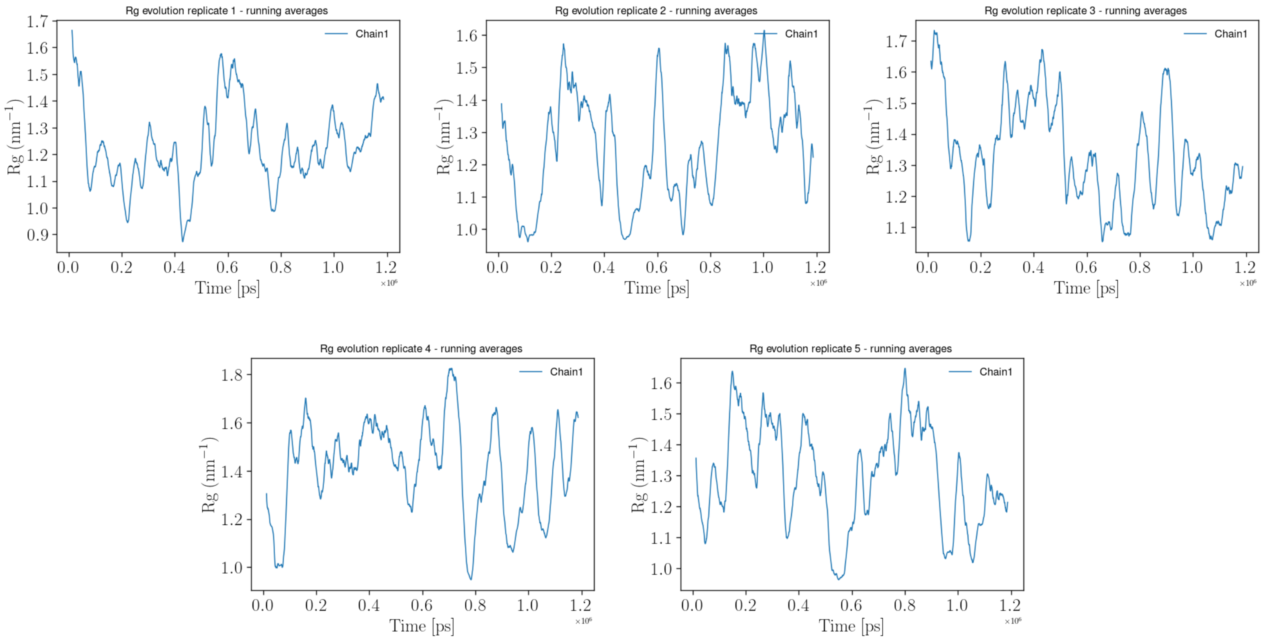

Figure S11: Evolution of the radius of gyration in the single-chain simulation using the A99SB-disp force field.

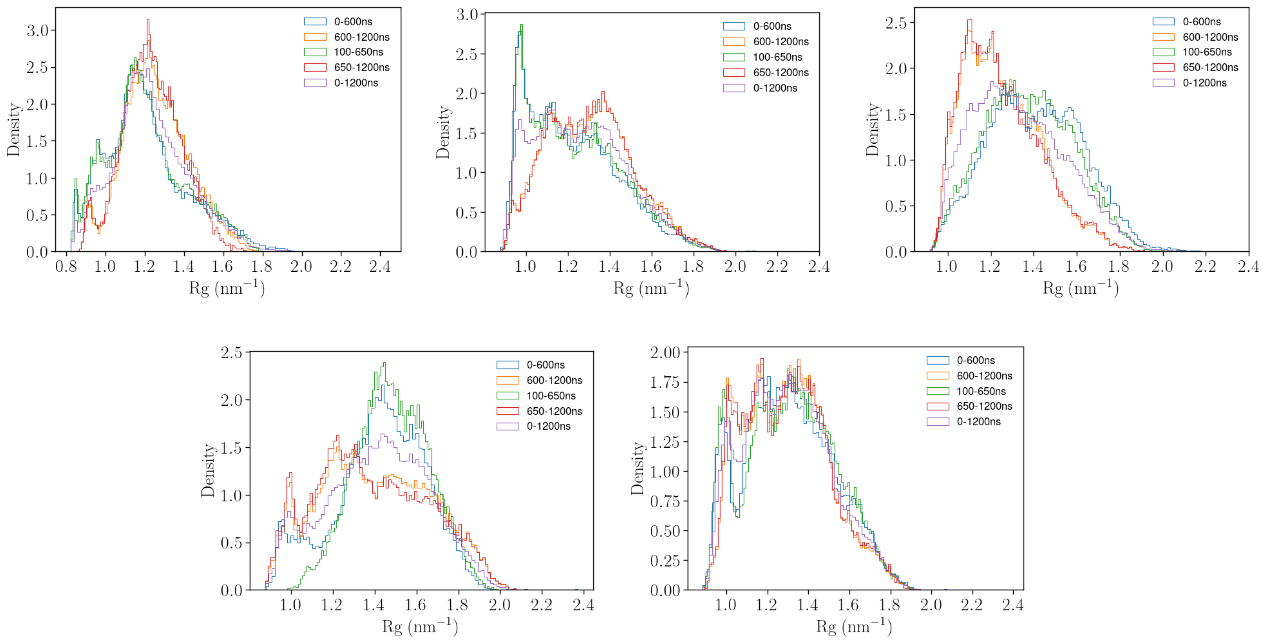

Figure S12: Distribution of radius of gyration for each replicate, for single-chain simulation using the A99SB-disp force field. Averages across different time-scales included to indicate the evolution of the distribution across time. From top left to bottom right: Replicate 1, 2, 3, 4, and 5, respectively.

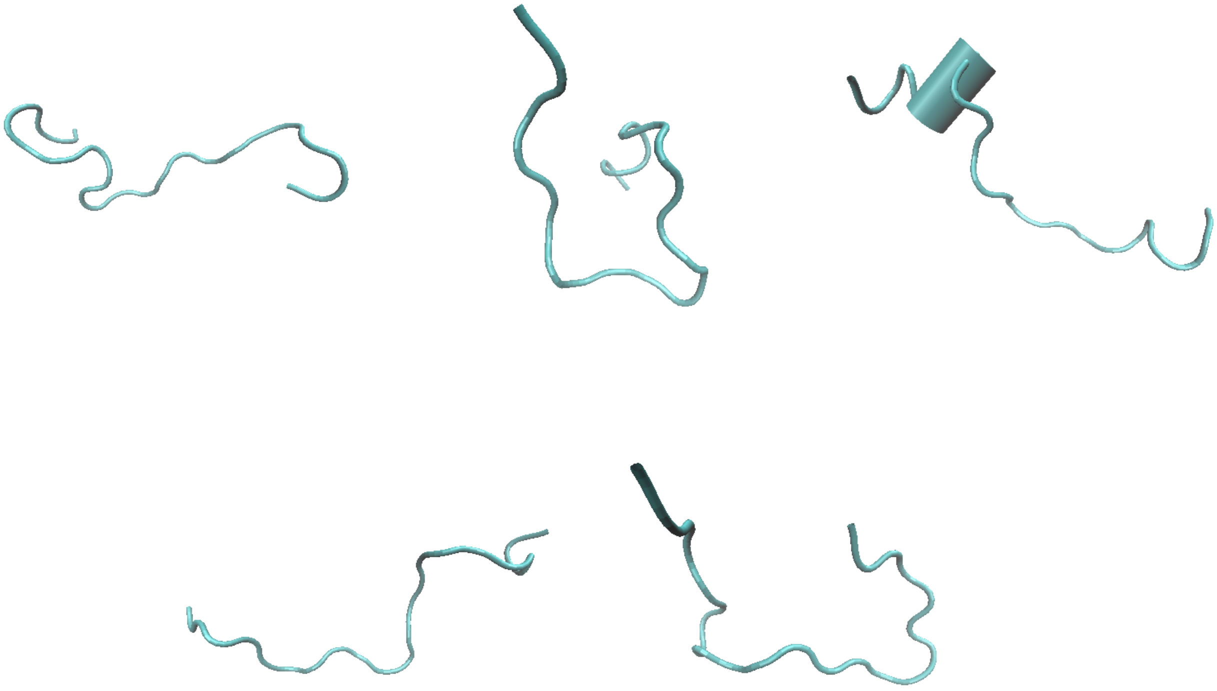

Figure S13: Snapshots from the last part of the trajectories of the single-chain simulation using the A99SB-disp force field. Going from top left to bottom right: replicate 1, 2, 3, 4, and 5, respectively.

Computed diffusion

Table S7: Diffusion and radius of gyration for each replicate in the simulation using the force field A99SB-disp. In this table, data is NOT corrected for finite-size effects or the water/deuterium difference. The number after the ± sign refers to the error of the linear fit to mean-square displacement. Error estimates are found by a block-averaging procedure.

Replicate	Trans. Diff. [Å^2/ns]	Rg [Å]
1	15.727 ± 0.042	12.316, err. est=0.4, std=1.9
2	16.151 ± 0.038	12.625, err. est=0.4, std=2.1
3	16.166 ± 0.046	13.324, err. est=0.5, std=2.1
4	15.770 ± 0.054	14.129, err. est=0.4, std=2.4
5	15.880 ± 0.038	12.979, err. est=0.5, std=2.1
Averages	15.939	13.075
STD	0.18626	0.62621

The lastly mentioned standard deviation in the above table is the standard deviation across replicate averages. The total standard deviation and error estimate (by block-averaging) was found to be 2.2 and 0.4 Å, respectively.

Computed simulation box volume: 1139.69, 1139.69, 1139.69, 1139.69, and 1139.68 nm^3 for replicate 1, 2, 3, 4, and 5, respectively. Average: 1139.69 nm^3.

Translational diffusion after fix of finite-size effects: 19.805 Å^2 / ns.

After adjusting for the fact that simulation was done in water, while experiment was performed in deuterium, correcting for viscosity difference between the two: 16.027 Å^2 / ns, standard deviation 0.15073

Using Hydropro As input to HYDROPRO, the viscosity and density of D2O is given, rather than using the values from simulation which used H2O (when not using HYDROPRO, this is adjusted for in post-processing). Snapshots from simulation were extracted every 100 ps. The following computed numbers (averaged across all replicates) were obtained:
Translational diffusion: 13.37, standard deviation 0.83 Å^2 / ns
Radius of gyration: 13.8 Å (Note that HYDROPRO assumes an atomic hydration layer of 1.1 Å in this computation).
No finite-size adjustment for diffusion is done in this case, as HYDROPRO is parametrized to directly compute properties from crystal structures.

7.5 10 mg/ml simulations

Convergence information

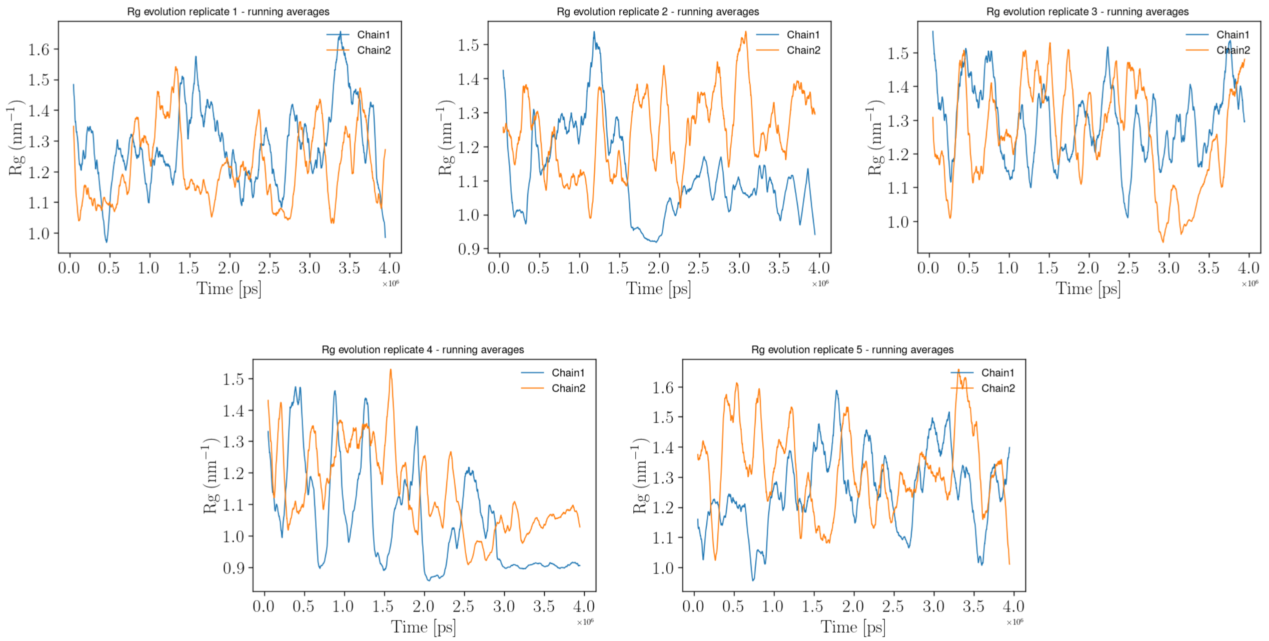

Figure S14: Evolution of the radius of gyration for the chains in the 10 mg/ml molecular dynamics simulation. Each replicate contains two chains. Running averages used for visual clarity, using a window of 1000, and with thinned-out data, using every 5th data point.

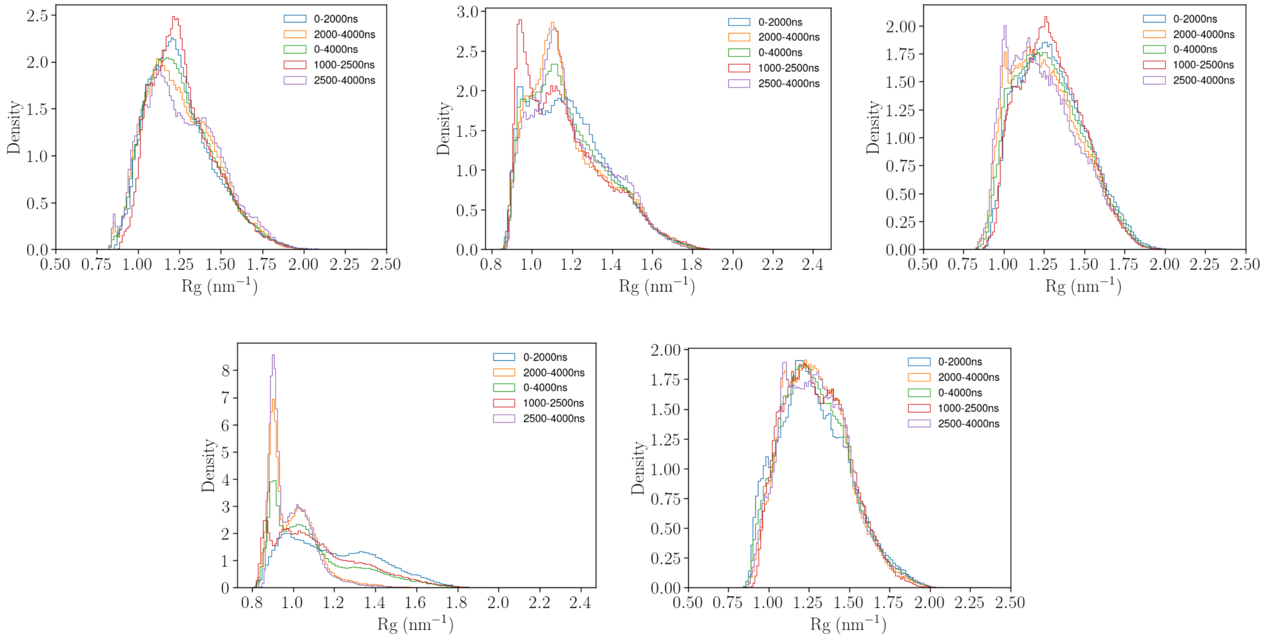

Figure S15: Distribution of radius of gyration for each replicate, for the 10 mg/ml simulations. Includes data from both chains present in each replicate. Averages across different time-scales included to indicate the evolution of the distribution across time. From top left to bottom right: Replicate 1, 2, 3, 4, and 5, respectively.

Average radius of gyration for each replicate (average of all chains in each replicate): 12.53, 11.84, 12.79, 10.99, and 12.89 Å. Total average of 12.21 Å.

Total standard deviation: 2.2 Å Total error estimate using block-averaging: 0.33 Å Standard deviation across replicates: 0.71 Å.

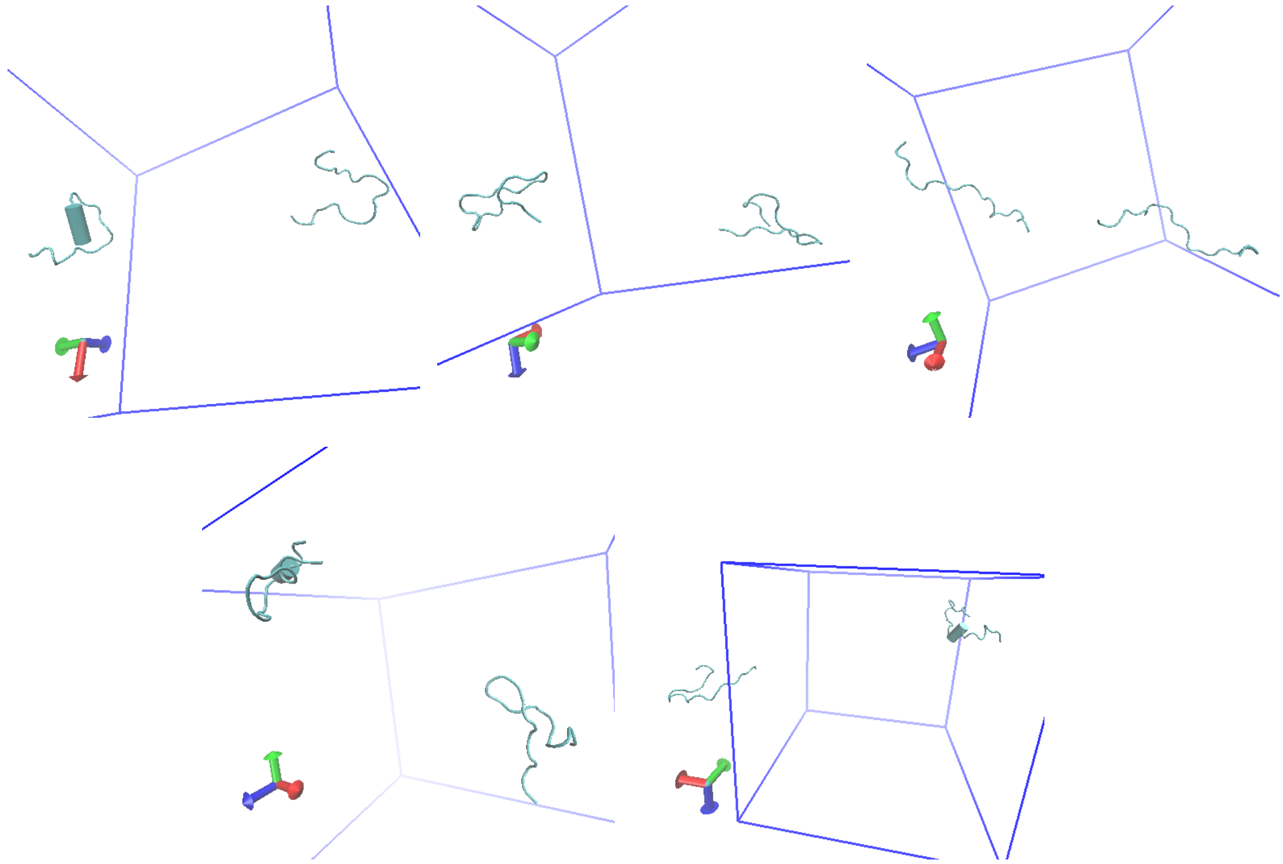

Figure S16: Last frames of the simulation for replicate 1, 2, 3, 4, and 5 going from top left to bottom right. Protein concentration of 10 mg/ml.

Numbers for the computation of diffusion parameters

Computed viscosity (averaged over all frames) for the replicates: 0.001965, 0.002057, 0.002055, 0.002144, and 0.002012 $kgm^{-1}s^{-1}$, respectively. Average: 0.002047 $kgm^{-1}s^{-1}$. Computed simulation box volume (averaged over all frames): 924.824, 924.822, 924.816, 924.83, 924.817 nm^3 for replicate 1, 2, 3, 4, and 5 respectively. Average: 924.822 nm^3

Translational diffusion after fix of finite-size effect: 18.005 ^{2} / ns

Table S8: Diffusion data for each chain in the 10 mg/ml simulations. In this table, data is NOT corrected for finite-size effects or the water/deuterium difference. The number after the ± sign refers to the error of the linear fit to mean-square displacement.

Replicate	Chain	Trans. Diff. [Å^2/ns]
1	1	15.175 ± 0.041
1	2	14.768 ± 0.039
2	1	14.807 ± 0.035
2	2	14.776 ± 0.044
3	1	15.069 ± 0.043
3	2	14.967 ± 0.044
4	1	15.094 ± 0.028
4	2	14.620 ± 0.042
5	1	14.956 ± 0.041
5	2	14.767 ± 0.045
Average	-	14.900
STD	-	0.16941

After adjusting for the fact that simulation was done in water, while experiment was performed in deuterium, correcting for viscosity difference between the two: 14.570 ^{2} / ns, standard deviation 0.13709

7.6 50 mg/ml simulations

Convergence information

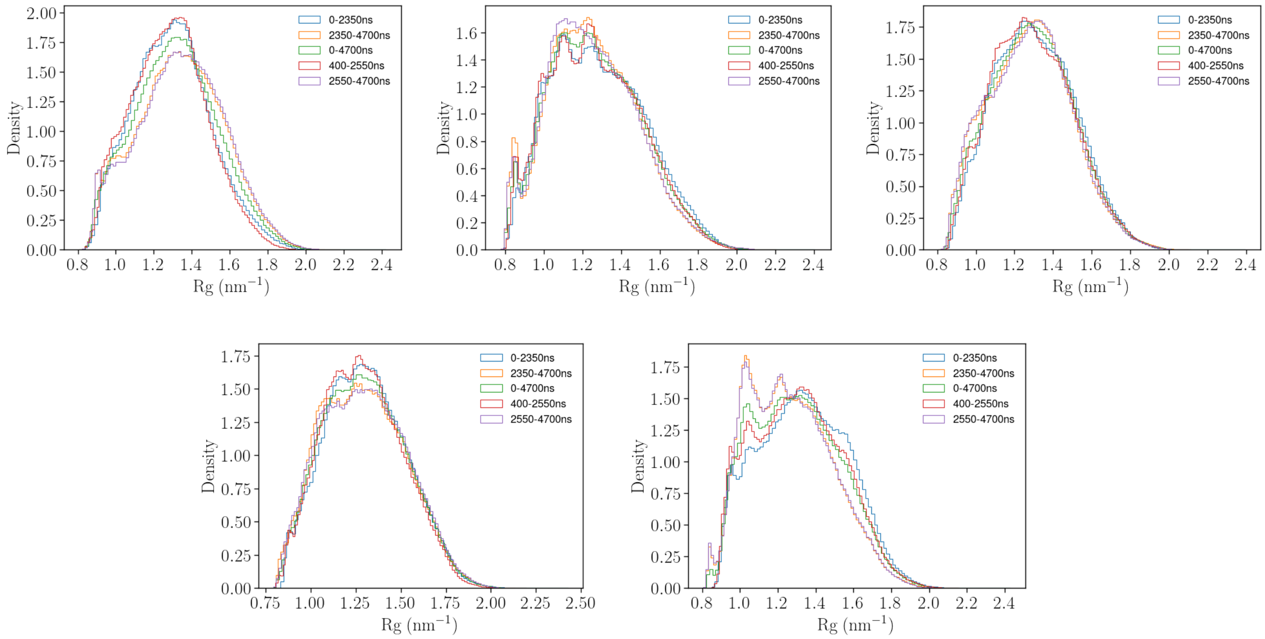

Figure S17: Distribution of radius of gyration for each replicate, for the 50 mg/ml simulations. Includes data from all chains present in each replicate. Averages across different time-scales included to indicate the evolution of the distribution across time.

Average radius of gyration for each replicate (average of all chains in each replicate):

13.12, 12.68, 12.93, 12.98, and 12.98 Å.

Average across replicates: 12.94 Å.

Error estimate, using block averaging for the total distribution: 0.1 Å

Standard deviation for the total distribution: 2.2 Å

Standard deviation across replicates: 0.14 Å.

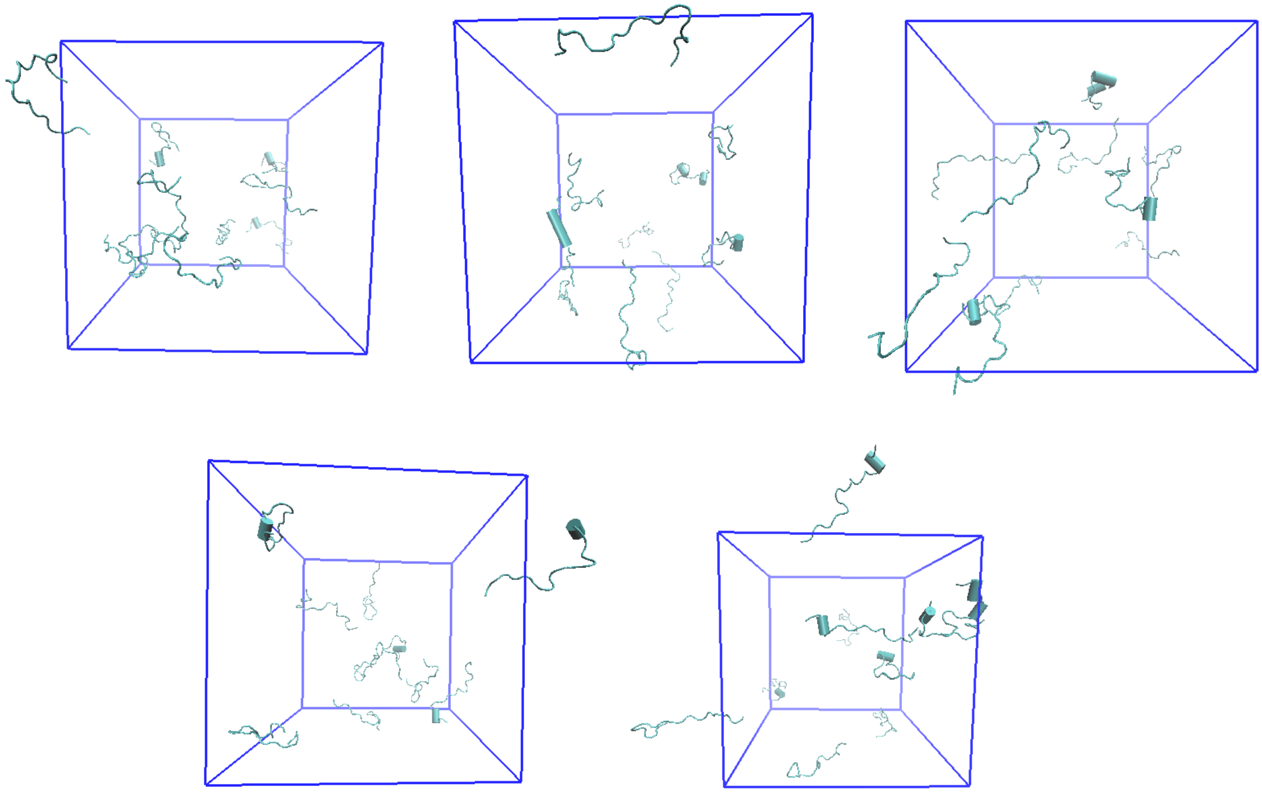

Figure S18: Last frames of the simulation for replicate 1, 2, 3, 4, and 5 going from top left to bottom right. Protein concentration of 50 mg/ml.

Clustering Analysis

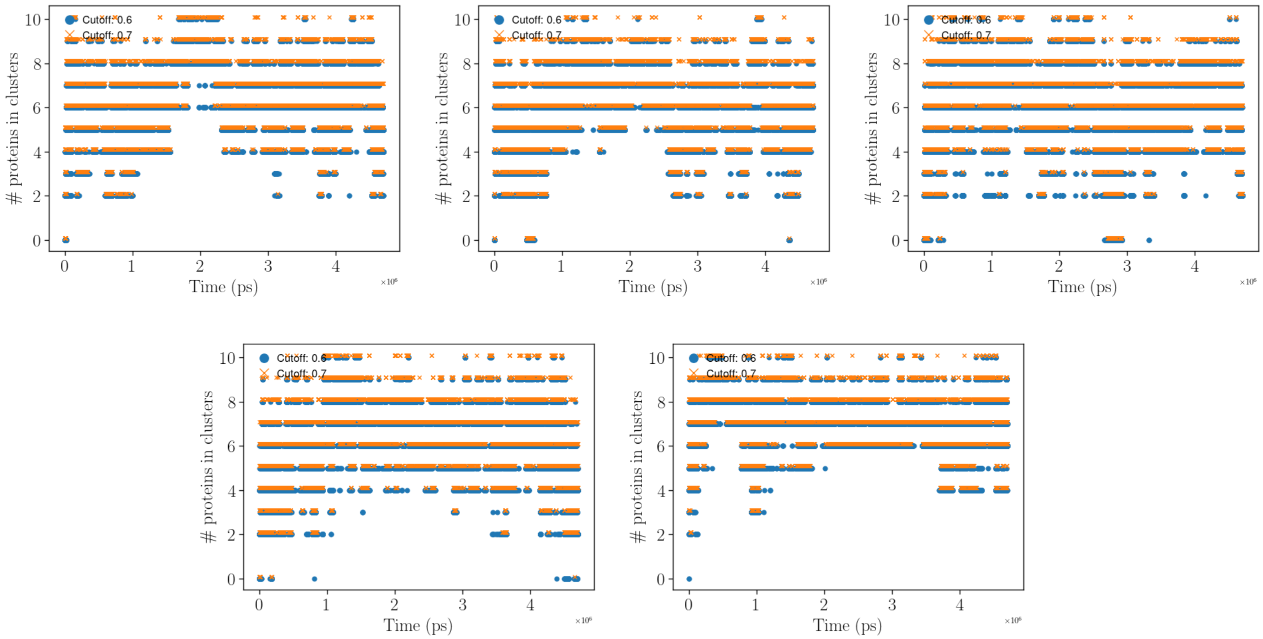

Figure S19: The number of proteins participating in any kind of cluster over the simulation trajectory. From top left to bottom right: Replicate 1, 2, 3, 4, and 5, respectively.

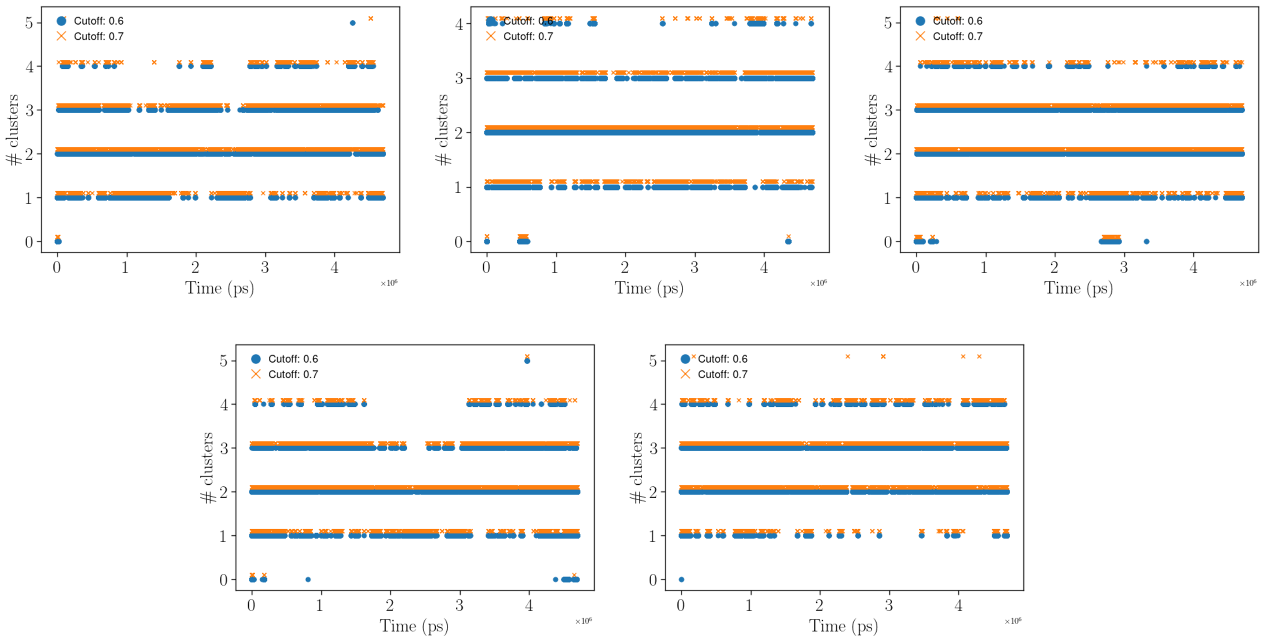

Figure S20: Number of transient clusters over the course of the simulation trajectory. From top left to bottom right: Replicate 1, 2, 3, 4, and 5, respectively.

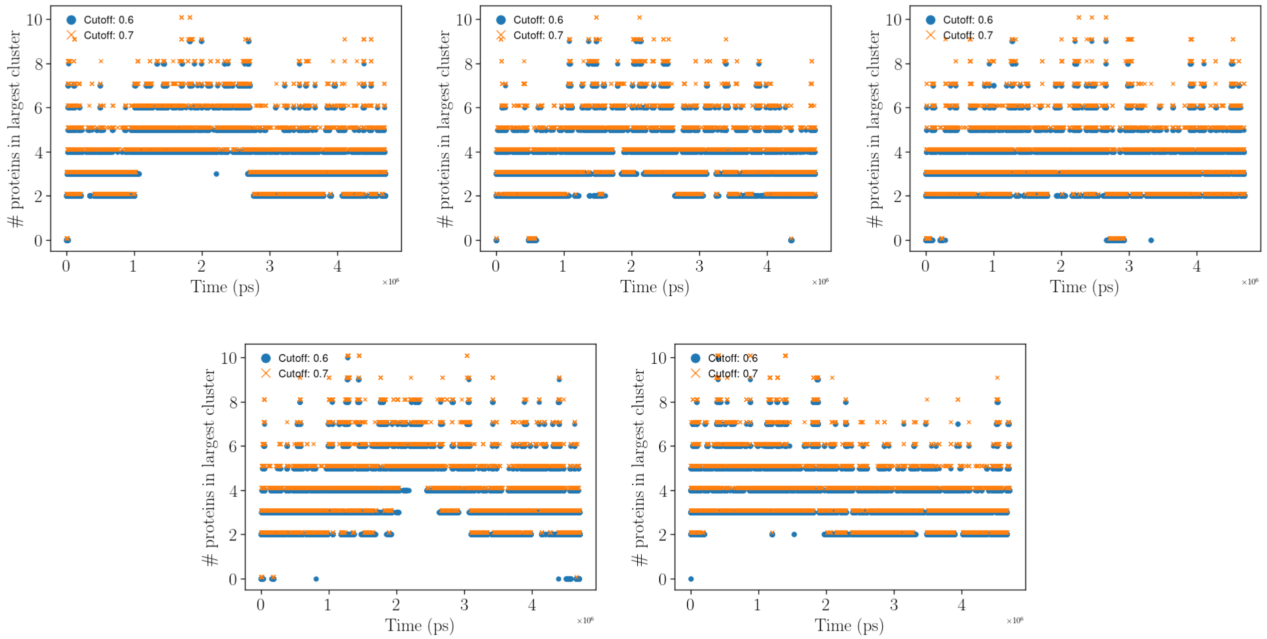

Figure S21: Number of proteins in the largest cluster in the replicate over the simulation trajectory. From top left to bottom right: Replicate 1, 2, 3, 4, and 5, respectively.

Table S9: Averages for different statistics of the transient clusters in the different replicates. "total # proteins in clusters" refers to the total amount of proteins in the replicates that participates in any cluster. The number in parenthesis is the cut-off used for defining the distance within two proteins are considered to be in a cluster together.

Replicate	1	2	3	4	5	Avg. over replicates
Avg. largest cluster (6 Å)	3.4	3.2	2.9	3.4	3.2	3.2
Avg. largest cluster (7 Å)	3.7	3.6	3.3	3.8	3.5	3.6
Avg. # clusters (6 Å)	2.2	2.0	2.2	2.1	2.6	2.2
Avg. # clusters (7 Å)	2.3	2.0	2.4	2.2	2.7	2.3
Avg. total # proteins in clusters (6 Å)	5.9	5.5	5.5	5.7	6.6	5.8
Avg. total # proteins in clusters (7 Å)	6.5	6.0	6.2	6.3	6.9	6.4

Numbers for the computation of diffusion parameters

Table S10: Translational diffusion for each chain in the 50 mg/ml protein concentration simulation. Computed using mean-square displacement and the Einstein relation. In this table, data is NOT corrected for finite-size effects or the water/deuterium difference. The number after the ± sign refers to the error of the linear fit to mean-square displacement.

Replicate	Chain	Trans. Diff. [$Å^2$/ns]
1	1	7.8221 ± 0.028
1	2	11.673 ± 0.041
1	3	11.828 ± 0.040
1	4	11.591 ± 0.038
1	5	11.212 ± 0.042
1	6	9.7226 ± 0.040
1	7	11.312 ± 0.044
1	8	10.100 ± 0.039
1	9	12.409 ± 0.042
1	10	8.6264 ± 0.033
2	1	12.690 ± 0.031
2	2	11.505 ± 0.036
2	3	12.374 ± 0.037
2	4	11.266 ± 0.039
2	5	8.5821 ± 0.029
2	6	10.116 ± 0.034
2	7	9.9161 ± 0.036
2	8	10.219 ± 0.039
2	9	11.739 ± 0.039
2	10	10.898 ± 0.034
3	1	9.8077 ± 0.035

3	2	11.351 ± 0.041
3	3	10.194 ± 0.038
3	4	11.979 ± 0.040
3	5	11.243 ± 0.043
3	6	11.267 ± 0.037
3	7	10.340 ± 0.041
3	8	11.407 ± 0.038
3	9	11.337 ± 0.039
3	10	10.578 ± 0.035
4	1	9.2242 ± 0.032
4	2	12.124 ± 0.044
4	3	8.6046 ± 0.033
4	4	11.436 ± 0.035
4	5	11.099 ± 0.039
4	6	10.132 ± 0.038
4	7	11.475 ± 0.045
4	8	11.353 ± 0.039
4	9	10.522 ± 0.034
4	10	11.032 ± 0.037
5	1	9.1837 ± 0.028
5	2	9.9705 ± 0.032
5	3	9.7827 ± 0.034
5	4	12.490 ± 0.042
5	5	9.3805 ± 0.032
5	6	9.6668 ± 0.029
5	7	9.3486 ± 0.035

5	8	11.3532 ± 0.045
5	9	11.779 ± 0.034
5	10	12.571 ± 0.030
Average	-	10.753
STD	-	1.1578

Per-replicate diffusion averages are 10.63 (STD=1.4), 10.93 (STD=1.2), 10.95 (STD=0.6), 10.70 (STD=1.0) and 10.55 (STD=1.3) ^{2} / ns for replicates 1, 2, 3, 4, and 5, respectively (not corrected for finite-size effects or water/deuterium difference).

Computed viscosity (averaged over all frames) for the replicates: 0.002685, 0.002495, 0.002568, 0.002522, and 0.002596 $kgm^{-1}s^{-1}$ respectively. Average: 0.002573 $kgm^{-1}s^{-1}$. Computed simulation box volume (averaged over all frames): 920.909, 920.919, 920.918, 920.921, and 920.921 nm^3 for replicate 1, 2, 3, 4, and 5, respectively. Average: 920.918 nm^3

Translational diffusion after fix of finite-size effects: 13.227 ^{2}/ns
After adjusting for the fact that simulation was done in water, while experiment was performed in deuterium, correcting for viscosity difference between the two: 10.704 ^{2} / ns, standard deviation 0.93693

For the individual replicates: Translational diffusion after fix of finite-size effects: 13.00, 13.30, 13.32, 13.07, and 12.92 ^{2} / ns for replicates 1, 2, 3, 4 and 5, respectively. After adjusting for the fact that simulation was done in water, while experiment was performed in deuterium, correcting for viscosity difference between the two: 10.52, 10.76, 10.78, 10.58, and 10.46 ^{2} / ns for replicates 1, 2, 3, 4 and 5, respectively.

Connecting diffusion and transient clusters

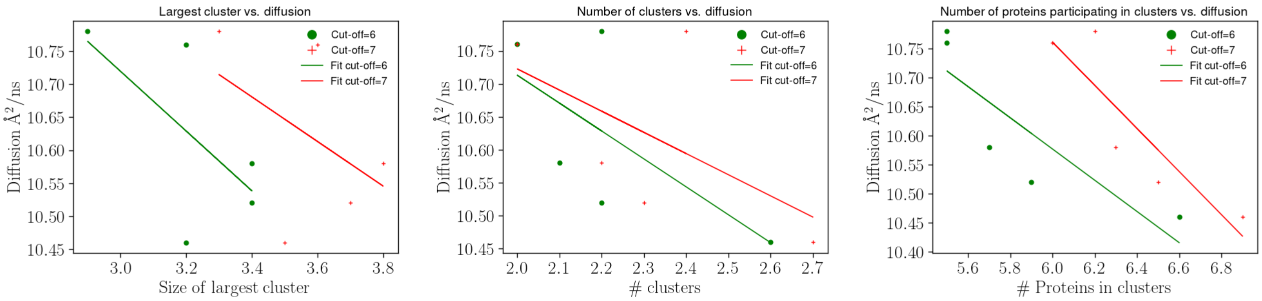

Figure S22: Scatter plots and linear regression of different metrics of clustering and the diffusion, for the different replicates in the 50 mg/ml simulation. **Left:** Largest cluster as cluster-metric, **middle:** Number of clusters as cluster-metric, **right:** Total number of proteins participating in any cluster as cluster metric.

Table S11: R^2 for the different linear regressions of cluster-metrics with diffusion.

Metric	R^2
Avg. largest cluster (6 Å)	0.42
Avg. largest cluster (7 Å)	0.20
Avg. # clusters (6 Å)	0.45
Avg. # clusters (7 Å)	0.33
Avg. total # proteins in clusters (6 Å)	0.73
Avg. total # proteins in clusters (7 Å)	0.79

7.7 Ramachandran plots

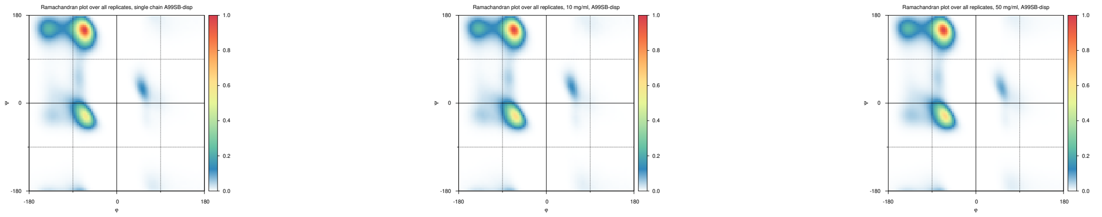

Figure S23: Ramachandran plots, using all replicates for each simulation case. Left: Single-chain simulation (A99SB-disp force field), Middle: 10 mg/ml protein concentration simulation, Right: 50 mg/ml protein concentration simulation.

References

(1) Goldsack, D. E.; Franchetto, R. The viscosity of concentrated electrolyte solutions. I. Concentration dependence at fixed temperature. *Can. J. Chem.* **1977**, *55*, 1062–1072.

(2) Cho, C.; Urquidi, J.; Singh, S.; Robinson, G. W. Thermal offset viscosities of liquid H2O, D2O, and T2O. *J. Phys. Chem. B* **1999**, *103*, 1991–1994.

(3) Goldsack, D. E.; Franchetto, R. The viscosity of concentrated electrolyte solutions. 11. Temperature dependence. *Can. J. Chem.* **1978**, *56*, 1442–1450.

(4) Goret, G.; Aoun, B.; Pellegrini, E. MDANSE: An Interactive Analysis Environment for Molecular Dynamics Simulations. *J. Chem. Inf. Model.* **2017**, *57*, 1–5.

(5) Henriques, J.; Skepö, M. Molecular Dynamics Simulations of Intrinsically Disordered Proteins: On the Accuracy of the TIP4P-D Water Model and the Representativeness of Protein Disorder Models. *J. Chem. Theory Comput.* **2016**, *12*, 3407–3415.

(6) Jephthah, S.; Pesce, F.; Lindorff-Larsen, K.; Skepö, M. Force Field Effects in Simulations of Flexible Peptides with Varying Polyproline II Propensity. *J. Chem. Theory Comput.* **0**, *0*, null.
